# Creation of X-linked Alport syndrome rat model with *Col4a5* deficiency

**DOI:** 10.1038/s41598-021-00354-y

**Published:** 2021-10-21

**Authors:** Masumi Namba, Tomoe Kobayashi, Mayumi Kohno, Takayuki Koyano, Takuo Hirose, Masaki Fukushima, Makoto Matsuyama

**Affiliations:** 1 Division of Molecular Genetics, Shigei Medical Research Institute, 2117 Yamada, Minami-ku, Okayama, 701-0202 Japan; 2 Division of Nephrology and Endocrinology, Faculty of Medicine, Tohoku Medical and Pharmaceutical University, Sendai, Japan; 3 Department of Endocrinology and Applied Medicine, Tohoku University Graduate School of Medicine, Sendai, Japan; 4 Shigei Medical Research Hospital, Okayama, Japan

**Keywords:** Biological techniques, Biotechnology, Developmental biology, Genetics, Diseases, Nephrology

## Abstract

Alport syndrome is an inherited chronic human kidney disease, characterized by glomerular basement membrane abnormalities. This disease is caused by mutations in *COL4A3*, *COL4A4*, or *COL4A5* gene. The knockout mice for *Col4*α*3*, *Col4*α*4*, and *Col4*α*5* are developed and well characterized for the study of Alport syndrome. However, disease progression and effects of pharmacological therapy depend on the genetic variability. This model was reliable only to mouse. In this study, we created a novel Alport syndrome rat model utilizing the rGONAD technology, which generated rat with a deletion of the *Col4*α*5* gene. *Col4*α*5* deficient rats showed hematuria, proteinuria, high levels of BUN, Cre, and then died at 18 to 28 weeks of age (Hemizygous mutant males). Histological and ultrastructural analyses displayed the abnormalities including parietal cell hyperplasia, mesangial sclerosis, and interstitial fibrosis. Then, we demonstrated that α3/α4/α5 (IV) and α5/α5/α6 (IV) chains of type IV collagen disrupted in *Col4α5* deficient rats. Thus, *Col4*α*5* mutant rat is a reliable candidate for the Alport syndrome model for underlying the mechanism of kidney diseases and further identifying potential therapeutic targets for human renal diseases.

## Introduction

Type IV collagen networks are the structural foundation for all basement membranes, consist of 6 different chains from COL4A1 to COL4A6 in mammals^1,2^. The 6 chains assemble into 3 types of heterotrimers (protomers) which have distinct tissue distribution and function. In the mammalian kidney, the glomerular basement membrane (GBM) requiring long-term maintenance of the glomerular filtration functions, is composed of α3/α4/α5 (IV) isoforms. Whereas α1/α1/α2 (IV) isoforms are found in all basement membranes, and α5/α5/α6 (IV) isoforms are detected in Bowman’s capsule (BC)^3^.

Alport syndrome is a basement membrane disorder, characterized by hereditary nephropathy that results in irreversible, progressive renal failure^4^. Alport syndrome is caused by mutations in *COL4A3*, *COL4A4*, or *COL4A5* gene encoding type IV collagen α3, α4, or α5 chains^5^. Defects in the *COL4A5* gene cause X-linked Alport syndrome, which accounts for about 80% of Alport syndrome^6^. And the remaining cases are associated with mutations in *COL4A3* or *COL4A4* genes. The pathological events of Alport syndrome are very similar, are found that lack of *COL4A3*, *COL4A4*, or *COL4A5* resulted in the disorganization of α3/α4/α5 (IV) network^1^.

In the past decades, several Alport syndrome animal models have been produced in mouse. The mutant mice for *Col4*α*3*, *Col4*α*4*, and *Col4*α*5* are developed and well characterized^7–12^. These model mice led important aspects of the renal disease. Whereas, there are some significant differences depend on genetic background in Alport syndrome model mice^9,13,14^. For example, 129X1/Sv or C57BL/6 was different patterns of disease progression in *Col4*α*3*^*-/-*^ mice^15^. Indeed, in the human patients, the disease progression and effects of pharmacological therapy were shown to genetic variability^6^. However, in spite of variabilities displayed in the genetic background, only a few mammal models (only mice and dogs^16^) have been established for Alport syndrome.

The laboratory rat (*Rattus norvegicus*) is a common experimental model for the human diseases and the drug testing^17–19^. For example, Wistar Kyoto (WKY) rat strain is known to be uniquely susceptible to crescentic glomerulonephritis among the strains tested^20,21^. Injection of isologous monoclonal antibodies caused anti-glomerular basement membrane antibody-induced glomerulonephritis (anti-GBM nephritis) in WKY rats^22,23^. Although there are several advantages comparing with mice, production of genetically engineered rat models has not yet been extensively proceeded during the past decades. The recent CRISPR/Cas9 system is the simplest for generating rats carrying a modified genome^24,25^, however, the standard method for genome-editing in mammals involves 3 major steps: isolation of zygotes from females, micromanipulation ex vivo, and transfer into pseudopregnant females. These 3 steps require extremely high level of technical expertise and its proficiency of the researchers as well as technicians. To simplify these complexed and laborious processes, we established to produce knock-out and knock-in mouse and rat with high efficiency, named as *i*-GONAD (mouse) and rGONAD (rat Genome-editing via Oviductal Nucleic Acids Delivery)^26–30^. The rGONAD method involves 2 key steps: an injection of the solution containing Cas9 protein, guide RNA and single strand DNA (ssDNA) into the oviduct, followed by electroporation. The rats that have undergone rGONAD are bred in routine way until birth. Moreover, rGONAD is highly efficient in both knock-out and knock-in rats^28,30^.

In the present study, we attempted to create Alport syndrome rat model using the rGONAD technology. We developed *Col4*α*5* deficient mutant rats, identical to the *Col4*α*5* G5X mutant mice^10^, which showed progressive glomerular disease and CKD phenotypes with renal fibrosis. And we investigated whether the mutant rats were applicable for the Alport syndrome model.

## Results

### Generation of Col4α5 deficient rats by the rGONAD method

On the basis of human mutations as described previously, we introduced novel *Col4α5* deficient rats with CRISPR/Cas9 and the rGONAD technology^28,30^. Tandem STOP codons were integrated into 27 bases after the first ATG in the rat *Col4α5* gene (Supplementary Fig. 1a). The mutation was verified by PCR followed by DNA sequencing, resulting to be expressed only 9 amino acids of COL4A5 at its N-terminus (Supplementary Fig. 1b,c). We detected no COL4A5 protein expression in *Col4α5* deficient male rats by immunofluorescence and Western blot analyses with monoclonal antibodies against the NC1 (C-terminus) domains of type IV collagens (See below; Figs. 7,8). In addition, the mutant rats which were integrated the other flame-shift tandem STOP codons (Col4α5 15 aa stop; Supplementary Fig. 2) and were deleted 56 bp including the first ATG (Col4α5 56 bp deletion; Supplementary Fig. 3), also generated, and revealed the same phenotype as Alport syndrome rats (Supplementary Fig. 2, 3). These data collectively indicate the successful generation of *Col4*α*5* deficient rats.

### Physiological analyses in Col4α5 mutants

**Figure 1:**
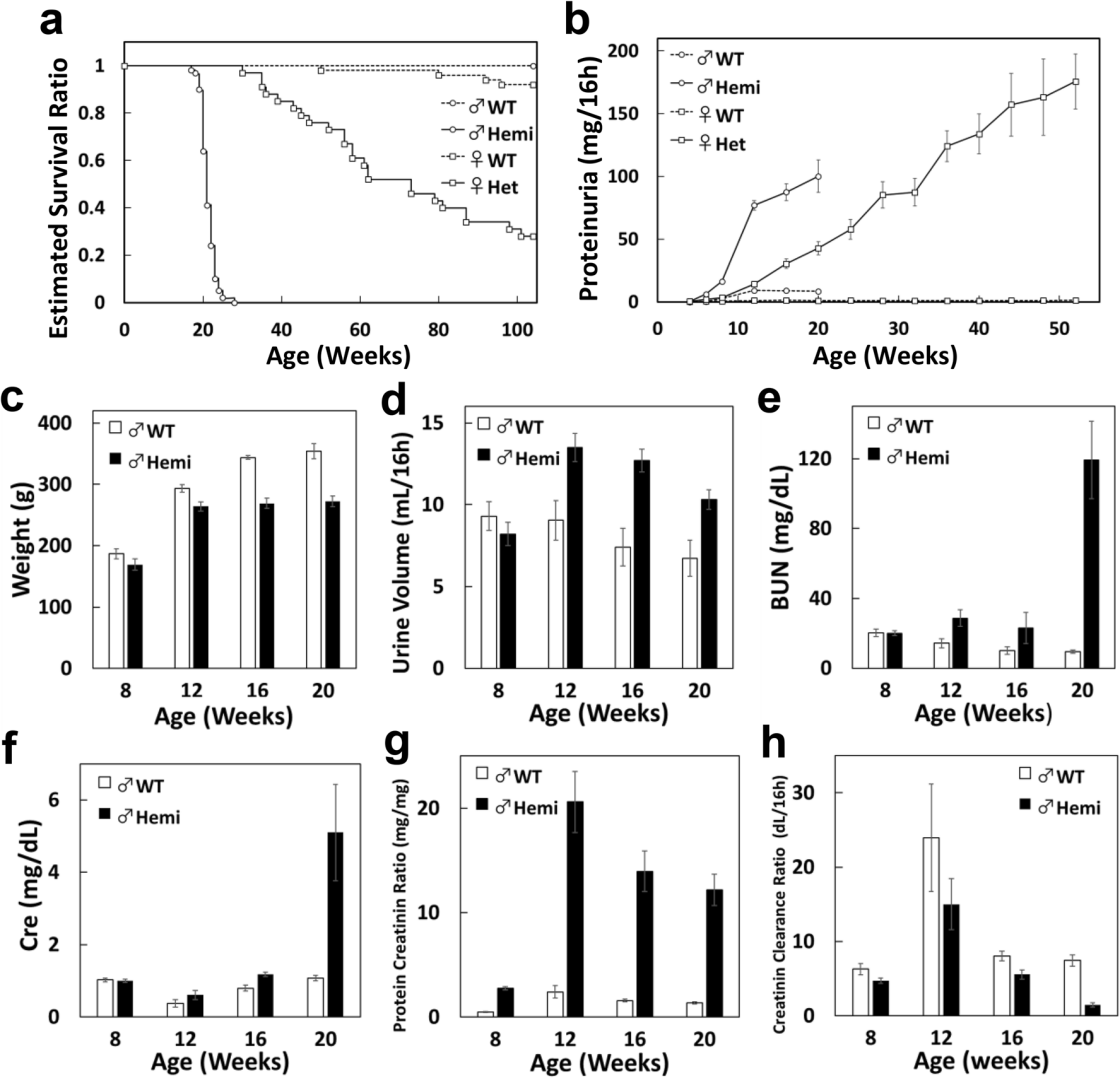
Physiological analyses in *Col4*α*5* mutant rats. **(a)** Estimated survival functions in wildtype (WT) and *Col4*α*5* mutant (Hemi; hemizygous males, het; heterozygous females) rats. **(b)** Proteinuria in wildtype and *Col4*α*5* deficient rats from 4 to 52 weeks of age. **(c–f)** Measurements of body weight **(c)**, urine volume **(d)**, blood urea nitrogen (BUN) of serum **(e)**, serum creatinine (Cre) **(f)**, protein creatinine ratio **(g)**, and creatinine clearance ratio **(h)** in wildtype and *Col4*α*5* mutant males from 8 to 20 weeks of age.

The following data are from the mutants rats shown in Supplementary Fig. 1a,b. *Col4*α*5* deficient rats were viable, fertile, and expected Mendelian ratios. However, all hemizygous mutant males died from 18 to 28 weeks of age (n = 58; Fig. 1a). Heterozygous mutant females died at 35 to 100 weeks of age, among them, approximately 30% of females survived over 100 weeks (n = 33, Fig. 1a). To assess of functional and histological abnormalities of the kidney, we measured the hematuria and proteinuria in *Col4*α*5* deficient rats. Hematuria was found from postnatal 21 days in hemizygous mutant males, and from 4 weeks of age in heterozygous mutant females (data not shown). Proteinuria was observed (about 6.0 mg/16 h) by 6 weeks of age in hemizygous mutant males (see Supplementary Table 1), then, increased > 20.0 mg/16 h in all of mutant males after 12 weeks of age (n = 35), and 62% of mutant females in 16 week of age (n = 28/45) (Fig. 1b). On the contrary, these phenomena were not detected in wildtype male and female rats in any week of age (Fig. 1b).

The mutant males showed decrease of body weight and increase of urine volume, compared with those of wildtype males (Fig. 1c,d). The levels of blood urea nitrogen (BUN) and creatinine (Cre) in serum highly increased in 20 weeks of age of mutant males (Fig. 1e,f). The protein/creatinine ratio was elevated in mutant males from 8 weeks of age, and creatinine clearance ratio was significantly lower in mutant males by 20 weeks of age (Fig. 1g,h).

### Renal histology in Col4α5 deficient rats

**Figure 2:**
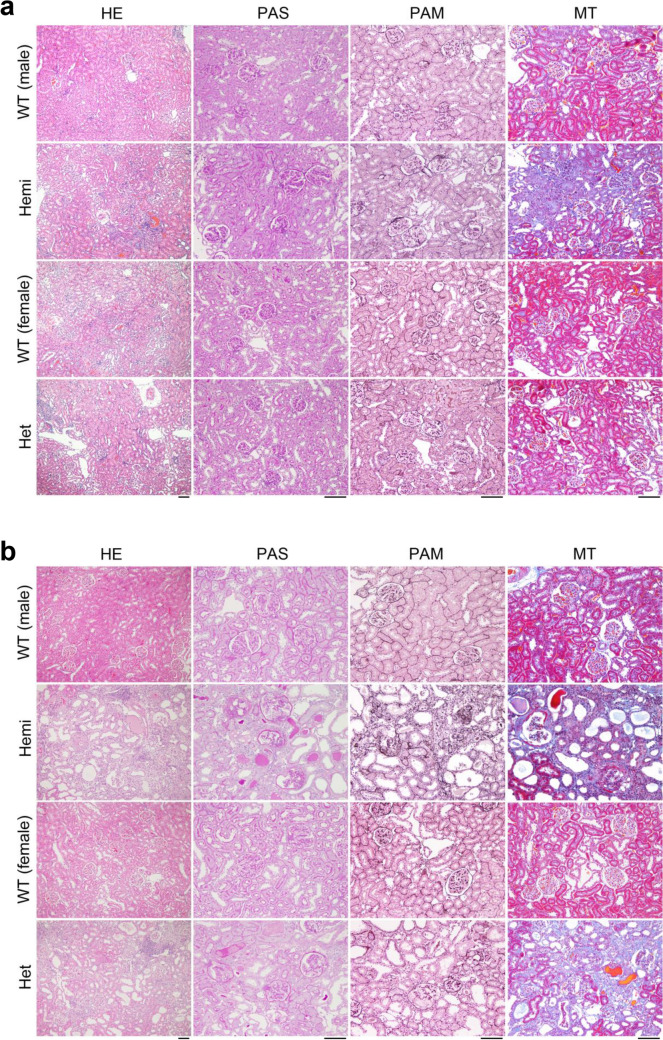
Histological analyses of *Col4*α*5* deficient kidneys. **(a,b)** Representative microscopic images in wildtype (WT) and *Col4*α*5* mutant (Hemi; hemizygous males, Het; heterozygous females) rats at 8 weeks **(a)** and 20 weeks **(b)** of age. These tissue sections were prepared and stained with hematoxylin and eosin (HE), periodic acid Schiff (PAS), periodic acid methenamine silver (PAM), and Masson trichrome (MT). *Scale bars*, 100 µm.

The kidney sections from wildtype and *Col4*α*5* deficient rats were stained with hematoxylin and eosin (HE), periodic acid Schiff (PAS), periodic acid methenamine silver (PAM), and Masson trichrome (MT) (Fig. 2). At 8 weeks of age, the glomeruli exhibited capillary tuft collapse in hemizygous mutant males (Fig. 2a). However, the kidney displayed overall sparing of the tubulointerstitium (Fig. 2a). By the 20 weeks of age in hemizygous mutant males, substantial numbers of glomeruli revealed the abnormalities, including parietal cell hyperplasia mimicking crescent formation, focal sclerosis, and with overall sparing of the tubulointerstitium (Fig. 2b, Supplementary Fig. 4). The kidney sections from heterozygous mutant females at 8–20 weeks of age displayed focal abnormalities of the glomeruli and tubulointerstitium (Fig. 2a,b, Supplementary Fig. 4).

**Figure 3:**
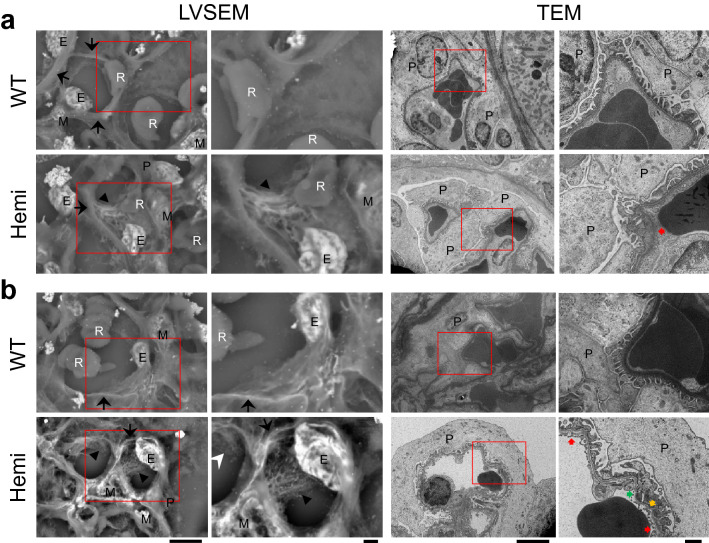
Electron photomicrographs of glomerular basement membranes in *Col4*α*5* mutant rats. **(a,b)** Representative low-vacuum scanning electron microscopy (LVSEM, left) and transmission electron microscopy (TEM; right) images in wildtype (WT) and *Col4*α*5* mutant (Hemi) males at 8 weeks **(a)** and 20 weeks **(b)** of age. Black and white arrowheads indicate the coarse meshwork structure of the GBM. Black arrows indicate cut side of the capillary walls. Red arrows indicate thickening of the GBM. Green arrows indicate thin patterns of the GBM. Yellow arrows indicate the splitting or fragmenting of the lamina densa. Red insets are revealed the higher magnification of left panels. E: Endothelial cells, M: Mesangial cells, P: Podocytes, R: Red blood cells. *Scale bars*, 5 µm (left), 1 µm (right).

To analyze the 3 dimensional ultrastructure of GBM, low-vacuum scanning electron microscopy (LVSEM) was performed, as described previously^31^. LVSEM revealed the coarse meshwork structure of the GBM with numerous pin-holes in hemizygous mutant males, in contrast to the smoothly arranged surface in wildtype males (Fig. 3, Supplementary Fig. 5). The transmission electron microscopy (TEM) image of the GBM in mutant males exhibited partial thickening from 8 weeks of age, and thickening of the GBM became accentuated and widely spread with increasing age (Fig. 3, Supplementary Fig. 5). Moreover, TEM also revealed substantial thinning of the GBM and split or fragmentation of the lamina densa by 20 weeks of age (Fig. 3, Supplementary Fig. 5).

### Renal glomerular and tubulointerstitial fibrosis in Col4α5 deficient rats

**Figure 4:**
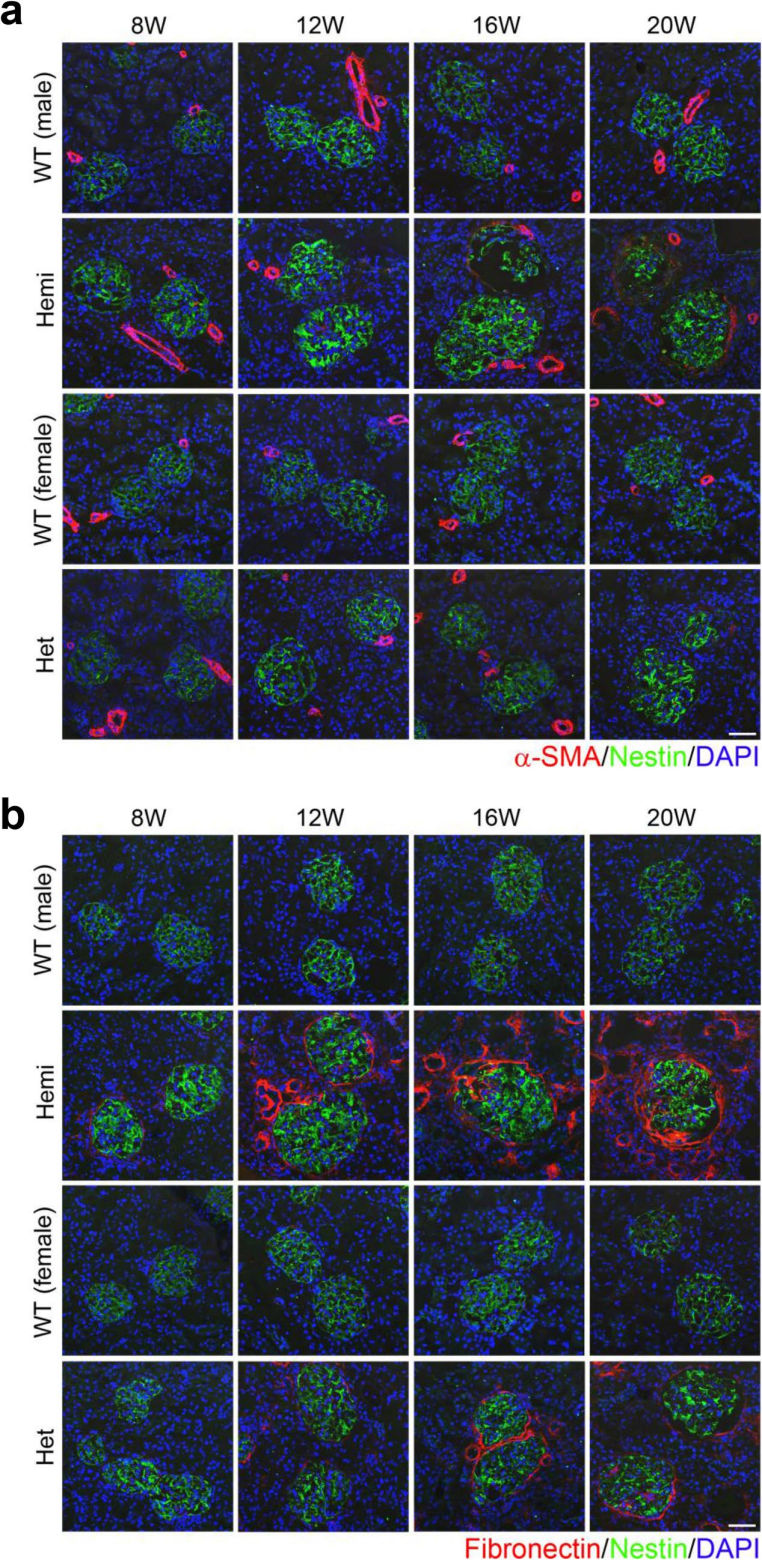
Renal fibrosis in *Col4*α*5* deficient rats. **(a,b)** Immunostaining of kidney sections with α-SMA **(a)** or fibronectin **(b)** (red), nestin (green; glomeruli), and DAPI (blue; nuclei) in wildtype (WT) and *Col4α5* mutant (Hemi; hemizygous males, Het; heterozygous females) rats from 8 to 20 weeks of age. *Scale bars*, 50 µm.

To evaluate fibrosis of the glomeruli and tubulointerstitium in *Col4*α*5* deficient rats, we examined kidney sections from wildtype and *Col4*α*5* deficient rats stained with antibodies specific for α-smooth muscle actin (α-SMA) and fibronectin (Fig. 4). Immunostaining showed that expression level of α-SMA, a maker of renal glomerular and tubulointerstitial fibrosis, was increased around the glomeruli from hemizygous mutant males when compared with those of wildtype littermates at 20 weeks of age, but it was not detected in the renal tubular epithelia (Fig. 4a). The α-SMA expression of kidney sections from the heterozygous females was rarely detectable at 20 weeks of age (Fig. 4a, Supplementary Fig. 6). In contrast, the expression level of fibronectin, a marker of pathological deposition of the extracellular matrix (ECM), was increased in 12 weeks of age of hemizygous mutant males (Fig. 4b). We then observed fibronectin expression in heterozygous mutant females at 12 weeks of age (Fig. 4b, Supplementary Fig. 7).

**Figure 5:**
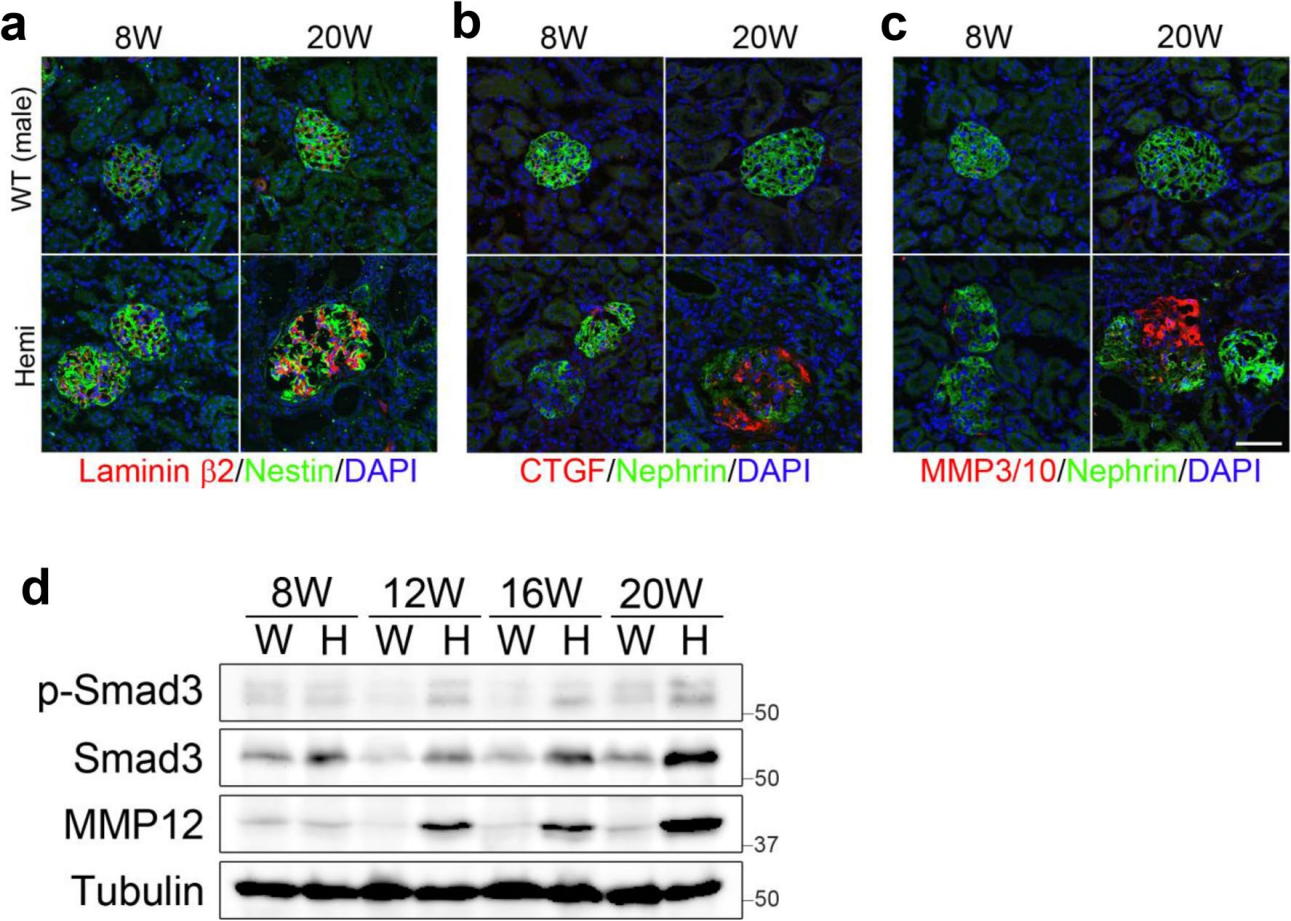
Immunostaining and Western blotting of the GBM in *Col4*α*5* deficient kidneys. **(a–c)** Immunofluorescence analyses of rat kidney sections with antibodies against **(a)**: Laminin β2 (red), Nestin (green; glomeruli); **(b)**: CTGF (red), Nephrin (green); **(c)**: MMP3/10 (red), Nephrin (green); and DAPI (blue; nuclei) in wildtype (WT) and *Col4α5* mutant (Hemi) male rats at 8 weeks (left) and 20 weeks (right) of age. *Scale bars*, 50 µm. **(d)** Detection of phospho- and total Smad3, and MMP12 by Western blot analyses of the kidneys in wildtype (W) and *Col4α5* mutant (H) male rats from 8, 12, 16, to 20 weeks of age. Tubulin was evaluated as an internal control.

Next, it has previously been reported that human, dog, and mouse Alport kidneys showed the progressive deposition in the GBM such as laminins^32,33^, and the progressive induction in glomeruli such as profibrotic cytokine TGFβ1^32^ and matrix metalloproteinase (MMPs)^34^. To characterize such possibilities in Alport model rats, we performed immunostaining and Western blotting of *Col4*α*5* deficient male kidneys for measuring the levels of these proteins. We first observed that an antibody to the laminin β2 chain exhibited intense staining of the GBM in *Col4*α*5* deficient males from 8 weeks of age (Fig. 5a, Supplementary Fig. 8a). To explore whether the TGFβ pathway was affected in *Col4*α*5* deficient rats, we examined levels of phospho-Smad3 which are the most critical mediators in TGFβ signaling pathway by Western blotting. We found that phosphorylation levels were increased in mutant male kidneys in comparison with those in wildtype (Fig. 5d). We then observed the protein expression of TGFβ downstream mediator CTGF^35^ in *Col4*α*5* deficient rats. Immunostaining analyses also showed the level of CTGF was increased in the GBM of mutant males by 12 weeks of age (Fig. 5b, Supplementary Fig. 8b). Moreover, we observed that an antibody to the MMP3/10 revealed intense staining of the GBM in *Col4*α*5* deficient males by 12 weeks of age (Fig. 5c, Supplementary Fig. 8c). In addition, we also detected increased MMP12 levels by Western blotting (Fig. 5d).

**Figure 6:**
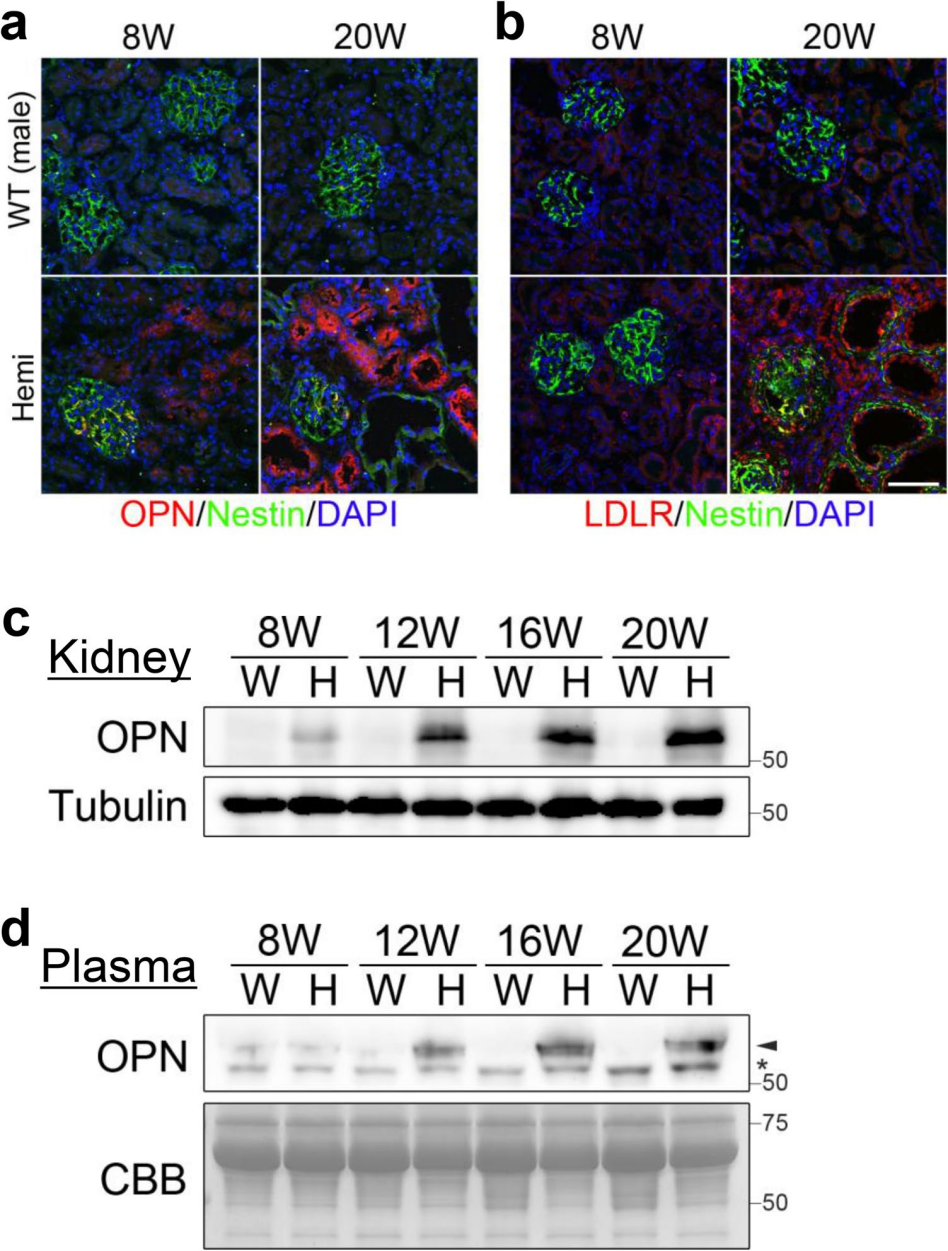
OPN and LDLR expressions in *Col4*α*5* deficient rats. **(a,b)** Immunostaining of kidney sections with **(a)** OPN or **(b)** LDLR (red), Nestin (green; glomeruli), and DAPI (blue; nuclei) in wildtype (WT) and *Col4α5* mutant (Hemi) male rats in wildtype (WT) and *Col4α5* mutant (Hemi) male rats at 8 weeks (left) and 20 weeks (right) of age. *Scale bars*, 50 µm. **(c,d)** Detection of OPN by Western blotting of the kidneys **(c)** or plasma **(d)** in wildtype (W) and *Col4α5* mutant (H) male rats from 8, 12, 16, to 20 weeks of age. Tubulin and CBB staining were evaluated as an internal control. Asterisk in **(d)** indicates non-specific signal.

In the recently report, osteopontin (OPN) and LDL receptor (LDLR) are highly expressed in renal tubules of Alport mice^36^. To investigate the expression of OPN and LDLR in Alport model rats, we performed immunostaining and Western blotting of *Col4α5* deficient male kidneys and/or plasma samples for measuring the levels of these proteins. Immunostaining analyses revealed the level of OPN was elevated in the renal tubules of mutant males from 8 weeks of age (Fig. 6a, Supplementary Fig. 9a). In addition, Western blotting also showed an increase of OPN expression in kidneys (Fig. 6c) and plasma (Fig. 6d) of *Col4α5* deficient males. In addition, LDLR expression was increased in the renal tubules of mutant males by 16 weeks of age by immunostaining (Fig. 6b, Supplementary Fig. 9b).

### The expression of type IV collagen α1-6 in Col4α5 deficient rats

The type IV collagen networks comprised of α1/α1/α2 (IV) (all basement membranes), α3/α4/α5 (IV) (GBM), and α5/α5/α6 (IV) (Bowman’s capsule) protomers were observed in the glomeruli. To examine whether localizations and/or levels of the proteins were changed in *Col4*α*5* deficient rats, we investigated *Col4*α*5* deficient rats with graded levels of the other’s type IV collagen α1, α2, α3, α4, and α6 (IV). First, we produced monoclonal antibodies that specifically recognize rat COL4A6 protein. We obtained rCol4A6 antibodies by injecting the antigen in *Col4a5* deficient males, but we could not obtain any specific antibody injected to wildtype rats at all. Immunoblot analysis revealed that the monoclonal anti-rCOL4A6, reacted specifically with a band corresponding to the position of the similar molecular weights in recombinant rCOL4A6 proteins, and only reacted with rCOL4A6, but not with other rCOL4 proteins (Supplementary Fig. 10).

**Figure 7:**
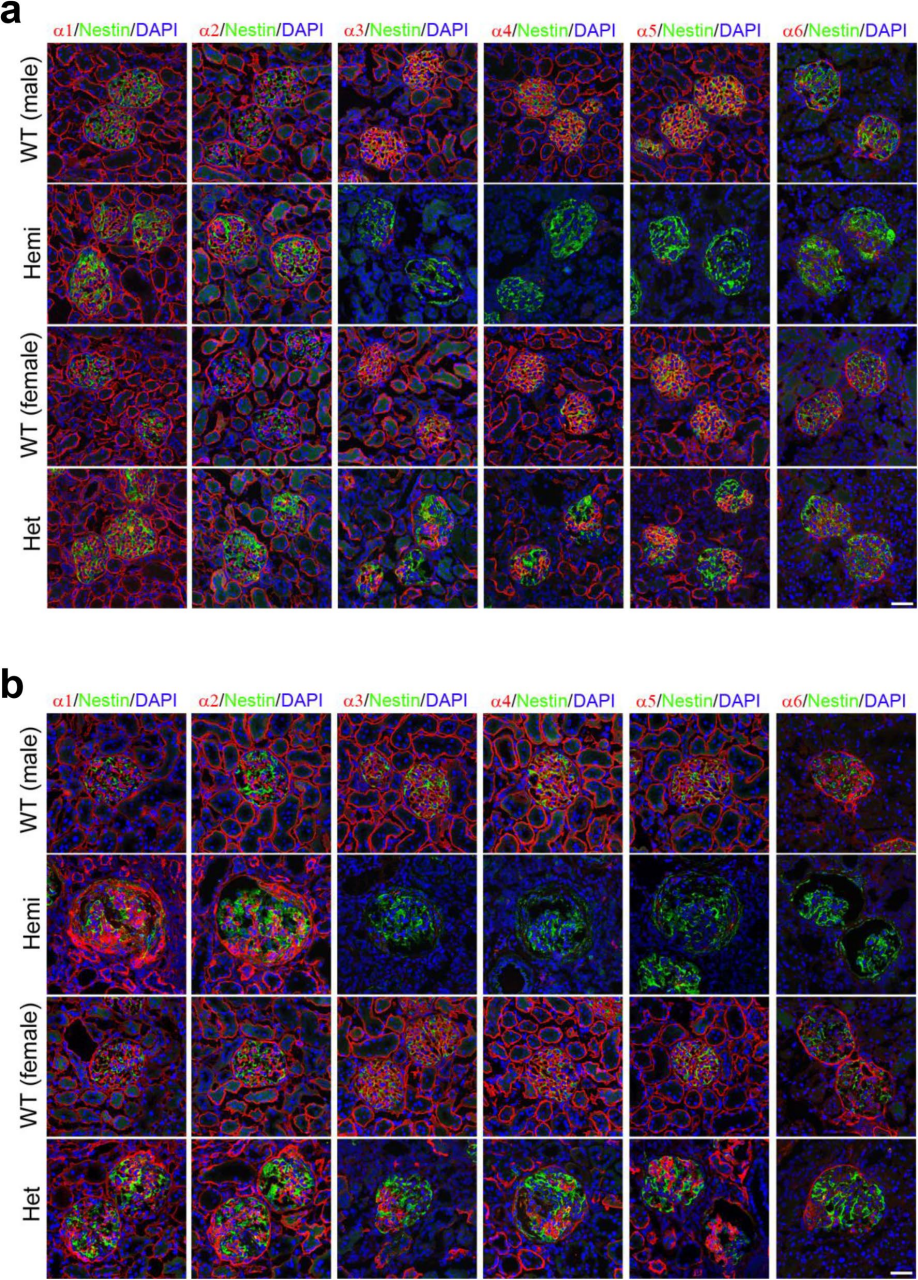
Type IV collagen distributions in *Col4*α*5* deficient kidneys. **(a,b)** Immunofluorescence analyses of kidney sections with antibodies against α1-6 (IV) (red), nestin (green; glomeruli), and DAPI (blue; nuclei) in wildtype (WT) and *Col4α5* mutant (Hemi; hemizygous males, Het; heterozygous females) rats at 8 weeks **(a)** and 20 weeks **(b)** of age. *Scale bars*, 50 µm.

To determine whether localization of type IV collagens of α1-6 (IV) in the kidney of *Col4*α*5* deficient rats changed, we performed the immunofluorescence analyses. At 8–20 weeks of age, COL4A5 expression was absent in hemizygous mutant males, and present in a mosaic pattern in heterozygous mutant females (Fig. 7a,b, Supplementary Fig. 11–14). The expressions of COL4A3, COL4A4, and COL4A6 were also absent in hemizygous mutant males, and present in a mosaic pattern in heterozygous mutant females (Fig. 7a,b, Supplementary Fig. 11–14). At 8 weeks of age, in contrast, expressions of COL4A1 and COL4A2 were present in *Col4α5* deficient rats (Fig. 7a, Supplementary Fig. 11). At 20 weeks of age, the kidney showed a strong accumulation of COL4A1 and COL4A2 proteins in both the GBM and Bowman’s capsule (Fig. 7b, Supplementary Fig. 14). These data suggest that α3/α4/α5 (IV) and α5/α5/α6 (IV) chains of type IV collagen disrupted in *Col4α5* deficient rats.

**Figure 8:**
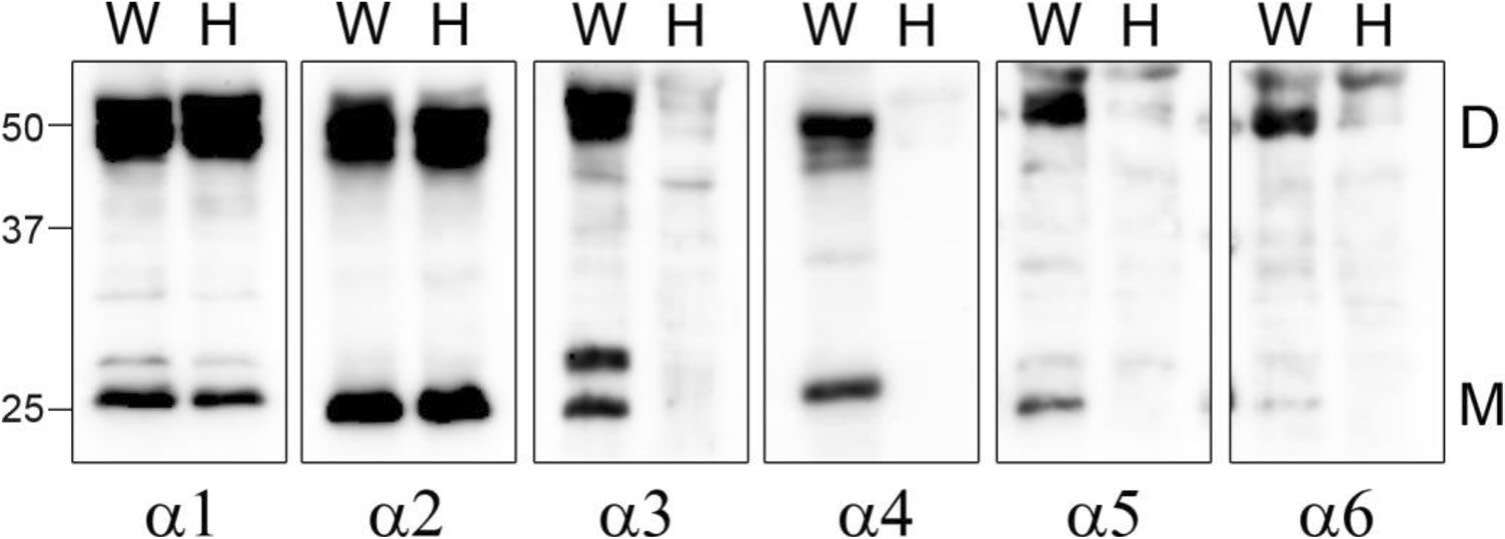
Western blot analyses of type IV collagen in *Col4*α*5* mutant kidneys. Collagenase-solubilized renal basement membranes from wildtype (W) and *Col4*α*5* mutant (H) males at 8 weeks of age were separated by SDS-PAGE and blotted with antibodies that the specifically recognized α1-6 (IV) NC1 domains. D: NC1 dimer, M: NC1 monomer.

To verify the results of immunostaining, we analyzed COL4 protein expressions of noncollagenous domains 1 (NC1) by Western blot analyses that were prepared from kidneys of wildtype and hemizygous mutant males (Fig. 8). There were no bands detectable NC1 domains of type IV collagen α3, α4, and α5 (IV), derived from α3/α4/α5 (IV) complexes, in kidney of *Col4α5* deficient males (Fig. 8). Then, the NC1 domains of type IV collagen α5 and α6 (IV), derived from α5/α5/α6 (IV) complexes, were also absent, whereas the blotting for the NC1 domains of type IV collagen α1 and α2 (IV), derived from α1/α1/α2 (IV) complexes, were present in hemizygous mutant males as well as wild type (Fig. 8). Therefore, these findings fully corroborate the results of immunofluorescence analyses.

## Discussion

The present study showed that we successfully created a novel Alport syndrome rat model with the rGONAD technology. *Col4*α*5* deficient rats revealed typical physiological, pathological, and also histological characteristics of Alport syndrome.

Occurrence of Alport syndrome is caused by mutations in *COL4A3*, *COL4A4*, or *COL4A5*^5^. Several mouse models in *Col4*α*3*, *Col4*α*4*, and *Col4*α*5* mutation have been known and characterized^7–11,14^. *Col4*α*3*^*-/-*^ or *Col4*α*4*^*-/-*^ mice are well used for studying treatments of Alport syndrome compared to *Col4*α*5*^*-/Y*^ mice^35,37–39^. Because, a survival ratio of *Col4*α*5*^*-/Y*^ mice was so diffusive ranged from 6 to 34 weeks^10^, in contrast, those of *Col4*α*3*^*-/-*^ or *Col4*α*4*^*-/-*^ mice were more uniform (from 13 to 26 weeks)^40,41^. In this study, *Col4*α*5* mutant males died at 18 to 28 weeks (about 95% from 20 to 26 weeks) of age. Thus, it is useful to study experiments for prolong the lifespan of *Col4*α*5* deficient rats for effects of the drug, food, and other factors.

In the present study, we are able to produce *Col4*α*5* mutant rats of WKY strain. Some number of glomeruli exhibited capillary tuft collapse at 8 weeks of age, and more glomeruli revealed abnormalities with crescent formation and focal sclerosis at the 20 weeks of age in *Col4*α*5* mutant males. The levels of BUN and Cre in serum highly increased in 20 weeks of age, and then died at around 24 weeks of age in hemizygous mutant males. These physiological and pathological features correspond to those of previously reported *Col4*α*3*^*-/-*^ with C57BL/6 mouse strain^13,15^. However, *Col4*α*3*^*-/-*^ with 129X1/Sv mouse strains showed much earlier progression of disease, died at around 12 weeks of age^13,40,42^. Therefore, comparison of a survival ratio between different strains of mice and rats should be carefully studied them, and further studies are required to draw a definite conclusion. In further study, other rat strains of *Col4*α*5* mutant using the rGONAD method can be expected.

In the past decades, there were several Alport syndrome models such as mouse and dog^16^. Using this Alport model rat, there are certainly advantages compared with those of mouse and dog. Rat is cheaper than dog to breed and maintain, and much reliable than dog to design genetic study because of much number of pups and much shorter gestational age. On the other hand, rat is more readily than mouse to measure the proteinuria level. Then, rat provides enough glomeruli, blood, and urine to proceed experiments that require significant amounts of protein, facilitating proteomic approaches to understanding glomerular disease mechanisms compared with mouse. Therefore, we strongly suggest that Alport syndrome model rat with the rGONAD method can be useful in variety of experimentation for underlying the mechanism of renal diseases.

The laboratory rat has long been recognized as a preferred experimental animal in wide areas of biomedical science^24,43^. In some situation, rat is considered as a more relevant model among mammals. For example, physiology of rat is greatly well documented, because its larger body size affords the opportunity for serial blood draw studies. In particular, blood pressure measurement by telemetry is easier to perform and more reliable in rat compared to smaller mouse^44,45^. Moreover, there are several rat strains in order to perform specific studies, i,e, SHR for hypertension^46^. Although genome engineering experiments of rat have yet not been extensively proceeded, it is readily applicable to produce the gene-editing mutant rats in *Col4*α*5* gene of the strains by the rGONAD method. The model rat might provide new insight into the study of the relationship between renal disease such as Alport syndrome and other blood pressure related diseases.

In conclusion, we have described creation of a novel rat model of X-linked Alport syndrome. This rat model should be available for the study of progressive renal failure having basement membrane abnormality. Thus, *Col4*α*5* mutant rat is a reliable candidate for Alport syndrome model animal for underlying the mechanism of renal diseases and further identifying potential therapeutic targets for human renal diseases.

## Methods

### Animals

WKY/NCrl rats were obtained from Charles River. The rats were kept with a 12:12-h light: dark cycle. They were given free access of drinking water and food. All animals were handled in strict accordance with good animal practice as defined by the relevant national and/or local animal welfare bodies. All animal works were approved by the Shigei Medical Research Institute Animal Care Committee (permission number: #17,006), and were performed in accordance with relevant guidelines and regulations. The manuscript was prepared according to the ARRIVE guidelines.

### Generation of type IV collagen α5 KO rats

Type IV collagen α5 deficient rats were produced by the rGONAD method as previously described^28,30^. Briefly, gene targeting strategy is designed to integrate tandem STOP codons into 27 bases after the first ATG in the rat *Col4α5* gene (Supplementary Fig. 1a). Guide RNAs were designed using CHOPCHOP (https://chopchop.cbu.uib.no/) (Supplementary Fig. 1a).

For the preparation of CRISPR/Cas9 reagents, Alt-R™ CRISPR-Cas9 system (Integrated DNA Technologies [IDT, Coralville, IA]) was used in accordance with the manufacturer’s protocol. Approximately 2–2.5 µl of electroporation solution was injected into the oviductal lumen from up-stream of ampulla using a micropipette. The electroporation was performed using a NEPA21 (NEPAGENE Co. Ltd., Chiba, Japan).

### Genotyping

Gene alteration was certified by PCR followed by DNA sequencing, as described previously^47^. Rat genomic DNA was isolated from ear-piece or tail. Genotyping was performed by PCR with the following primers (see also Supplementary Fig. 1A): rCol4a5-fw, (5′-GCTCTCTTCCCAATAACCCCT-3′), rCol4a5-rv, (5′-CAATTTTGACTTCCCTGGCCA-3′).

### Urine and blood parameters

Urine samples were collected for 16 h by placed rats in metabolic cages individually, every 4 weeks over 52 weeks (see Supplementary Table 1). Proteinuria level was measured by a modified method using 3% sulfosalicylic acid. Blood samples (n = 6 each) were collected from the tail and centrifuged at 3000 g for 5 min to obtain blood serum. Blood urea nitrogen (BUN) of serum was measured using Colorimetric Detection Kit (Arbor Assays, Michigan, USA). Creatinine (Cre) levels of serum or urine were measured Jaffe’s method (FUJIFILM Wako Pure Chemical Corporation, Osaka, Japan). Creatinine clearance ratio was calculated by (urine creatinine [mg/dl] x urine volume [dl])/serum creatinine [mg/dl]. All measurements were carried out according to manufacturer’s recommended protocols.

### Histological analyses

Rat kidneys were soaked in 10% buffered neutral formalin at least overnight, and embedded in paraffin after dehydrated. The embedded kidneys were sliced 1–2 µm thickness. These slides were stained with hematoxylin and eosin (HE), periodic acid Schiff (PAS), periodic acid methenamine silver (PAM) and Masson's trichrome (MT) by standard methods.

### Low vacuum scanning electron microscopy (LVSEM) and transmission electron microscopy (TEM)

Under LVSEM, 3 dimensional ultrastructure of the GBM of Alport syndrome, the kidney was examined as described previously^31^. In brief, renal paraffin sections of 4 µm thickness were stained with PAM. The sections on the slides were directly observed without a cover clip, with LVSEM (Hitachi TM4000; Hitachi Co. Ltd., Tokyo, Japan) at acceleration voltage of 15 kV with 30 Pa.

TEM was performed as described previously^48^. Ultrathin epoxy resin sections were prepared and observed using an H-7600 electron microscope (Hitachi Co. Ltd.).

### Preparation of recombinant proteins

His-tagged (for production of antibody) and MBP-tagged (for immunoblotting) NC1 domains of type IV collagen α1-6 (V) were expressed in BL21-CodonPlus-RP (Agilent Technologies, Santa Clara, CA) transformed with pET-28a (Invitrogen) and pMAL (New England Biolabs, Beverly, MA), respectively. Each His or MBP fusion protein was purified through affinity chromatography with TALON metal affinity resin (Clonetech, Palo Alto, CA) or with amylose resin (New England Biolabs), respectively.

### Antibodies

We produced rat monoclonal rCOL4A6, as described previously^49,50^. Briefly, the antigen emulsion was injected to *Col4a5* deficient males. The treated rats were sacrificed 21 days after the injection, and the lymphocytes were fused with SP2/0-Ag14 myeloma cells. After the cell fusion, culture supernatants were screened to confirm positive clones by solid-phase enzyme-linked immunosorbent assay (ELISA).

The following primary antibodies were used: rat monoclonal type IV collagen α1(IV) (H11), α2 (IV) (H22), α3 (IV) (H31), α4 (IV) (H43), α5(IV) (H52) (In our institute)^3^; Tubulin (T9026), α-SMA (1A4) (Sigma, St. Louis, MO); phospho-Smad3 (ab52903), Fibronectin (ab6328), LDL receptor (ab52818), MMP12 (ab52897) (Abcam, Cambridge, UK); Smad3 (#9523, Cell Signaling Technology, Beverly, MA); Nephrin (GP-N2, Progen, Germany); Nestin (66259-1-Ig, proteintech, Chicago, IL); Laminin β2 (MAB2066), OPN (AF808) (R&D systems Minneapolis, MN); CTGF (sc-373936), MMP3/10 (sc-374029) (Santa Cruz Biotechnology, Santa Cruz, CA); and MBP (New England Biolab). Primary antibodies were detected using species-specific secondary antibodies conjugated to either Alexa Fluor 488 or 555 (Molecular probes).

### Tissue extract preparation and immunoblotting

Rat kidneys and blood plasma were homogenized directly in SDS-PAGE sample buffer. For collagenase-solubilized renal basement membranes, rat kidneys were homogenized and incubated at 37 °C for 4 h with 1 mg kidney lysates and 200 µg (1000 U) of collagenase (Brightase-C; Nippi, Okayama, Japan) in digestion buffer (20 mM HEPES, 10 mM CaCl_2_) with protease inhibitor cocktail (Nacalai, Kyoto, Japan). Protein concentrations for tissue extracts were determined by Coomassie Brilliant Blue staining by SDS-PAGE gels. The lysates were loaded, transferred, and subjected to Western blotting with specific antibodies as described previously^51^.

### Immunofluorescence

Immunofluorescence analyses were examined, as described previously^52^. Briefly, rat kidneys were immersed in OCT compound, and snap-frozen in liquid nitrogen vapor. Subsequently, kidneys were sliced 5 µm cryostat sections and placed on slides. After dehydration by acetone, blocked in 5% (v/v) donkey serum for 30 min at room temperature. Sections were then incubated with primary antibodies overnight at 4 °C followed by PBS wash and incubated with appropriate secondary antibodies for 1 h at room temperature. DNA was also stained with 1 µg/ml DAPI. Fluorescence images were obtained by confocal microscopy (FV1200, Olympus, Japan).

## Supplementary Information

Supplementary Information.

## Data Availability

All data generated and analyzed during this study are included in this published article and its Supplementary Information.
