# Publisher Correction: Creation of X-linked Alport syndrome rat model with *Col4a5* deficiency

**DOI:** 10.1038/s41598-021-01409-w

**Published:** 2021-11-08

**Authors:** Masumi Namba, Tomoe Kobayashi, Mayumi Kohno, Takayuki Koyano, Takuo Hirose, Masaki Fukushima, Makoto Matsuyama

**Affiliations:** 1 Division of Molecular Genetics, Shigei Medical Research Institute, 2117 Yamada, Minami‑ku, Okayama, 701‑0202 Japan; 2 Division of Nephrology and Endocrinology, Faculty of Medicine, Tohoku Medical and Pharmaceutical University, Sendai, Japan; 3 Department of Endocrinology and Applied Medicine, Tohoku University Graduate School of Medicine, Sendai, Japan; 4 Shigei Medical Research Hospital, Okayama, Japan

Correction to: *Scientific Reports* 10.1038/s41598-021-00354-y, published online 21 October 2021

The Supplementary Information published with this Article contained errors where there the Figure titles were incorrect in the Contents and the legends of Supplementary Figures S2, S3, and S9.

“Supplementary Figure S2. Analyses of “Col4*α* 15aa stop” mutant rats”

now reads:

“Supplementary Figure S2. Analyses of “Col4*α*5 15 aa stop” mutant rats”

“Supplementary Figure S3. Analyses of “*Col4α5* 56bp deletion” mutant rats”

now reads:

“Supplementary Figure S3. Analyses of “*Col4α5* 56 bp deletion” mutant rats”

“Supplementary Figure S9. OPN and LRLR expressions in *Col4α5* deficient rats”

now reads:

“Supplementary Figure S9. OPN and LDLR expressions in *Col4α5* deficient rats”

Additionally, in the legend of Supplementary Figure S3,

“(a) Direct sequence of mutation.”

now reads:

“(a) Schematic diagram of *Col4α5* 56 bp deletion.”

The original Supplementary Information file is provided below.

These errors have now been corrected in the Supplementary Information file that accompanies the original Article.

## Supplementary Information

Supplementary Information.

