# Supplementary material for: Creation of X-linked Alport syndrome rat model with Col4a5 deficiency
Source: Sci Rep. 2021 Oct 21;11:20836. doi: 10.1038/s41598-021-00354-y (PMC8531394; doi:10.1038/s41598-021-00354-y)
Supplement: Supplementary file 1 — Supplementary Information. [file 41598_2021_354_MOESM1_ESM.pdf]

Creation of X-linked Alport Syndrome Rat Model with *Col4a5* Deficiency

Masumi Namba[1], Tomoe Kobayashi[1*], Mayumi Kohno[1*], Takayuki Koyano[1], Takuo Hirose[2, 3], Masaki Fukushima[1, 4], Makoto Matsuyama[1].

[1] Division of Molecular Genetics, Shigei Medical Research Institute, Okayama, Japan.

[2] Division of Nephrology and Endocrinology, Faculty of Medicine, Tohoku Medical and Pharmaceutical University, Sendai, Japan.

[3] Department of Endocrinology and Applied Medicine, Tohoku University Graduate School of Medicine, Sendai, Japan.

[4] Shigei Medical Research Hospital, Okayama, Japan.

* These authors contributed equally to this work.

Corresponding to Makoto Matsuyama;

Division of Molecular Genetics, Shigei Medical Research Institute.

2117 Yamada, Minami-ku, Okayama 701-0202, Japan.

TEL: +81-86-282-3113

E-mail: matsuyama@shigei.or.jp

Running title: Alport syndrome model in rats

Supplementary Information

Contents:

Supplementary Figure S1. Production of *Col4α5* mutant rats

Supplementary Figure S2. Analyses of “Col4*α5* 15 aa stop” mutant rats

Supplementary Figure S3. Analyses of “*Col4α5* 56 bp deletion” mutant rats

Supplementary Figure S4. Histological analyses of *Col4*α*5* deficient kidneys

Supplementary Figure S5. Electron photomicrographs of glomerular basement membranes in *Col4*α*5* mutant rats

Supplementary Figure S6. Renal fibrosis in *Col4*α*5* deficient rats

Supplementary Figure S7. Renal fibrosis in *Col4*α*5* deficient rats

Supplementary Figure S8. Immunostaining of the GBM in *Col4α5* deficient kidneys

Supplementary Figure S9. OPN and LDLR expressions in *Col4α5* deficient rats

Supplementary Figure S10. Characterization of an antibody specifically recognized type IV collagen

Supplementary Figure S11. Type IV collagen distributions in *Col4*α*5* deficient kidneys at 8 weeks of age

Supplementary Figure S12. Type IV collagen distributions in *Col4*α*5* deficient kidneys at 12 weeks of age

Supplementary Figure S13. Type IV collagen distributions in *Col4*α*5* deficient kidneys at 16 weeks of age

Supplementary Figure S14. Type IV collagen distributions in *Col4*α*5* deficient kidneys at 20 weeks of age

Supplementary Figure S15. Scan images for Western blotting

Supplemental Figure S16. Scan images of gel electrophoresis

Supplementary Table S1. Proteinuria in *Col4a5* deficient rats

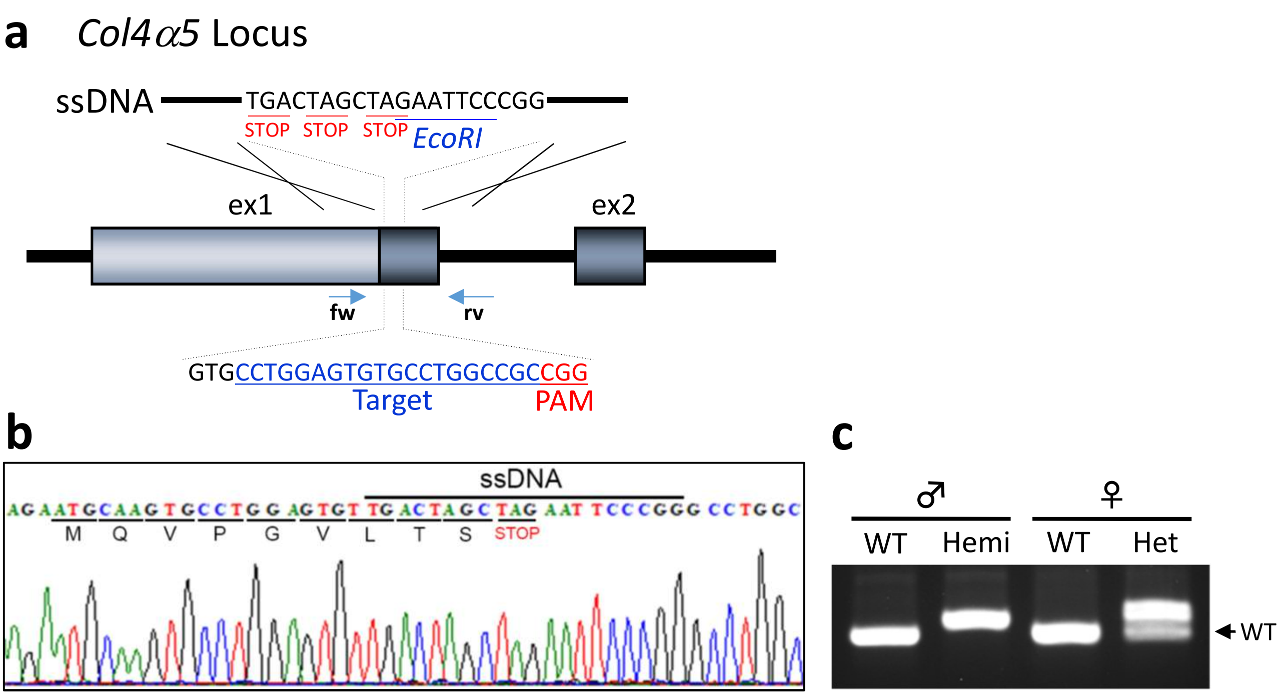

d

Stain	Treated female	pregnant mice	Newborns	modified allele	deletion	ssODN insertion
WKY	16	14	100	37 (37%)	29 (29%)	8 (8%)

Supplementary Figure S1. Production of *Col4α5* mutant rats

(a) Schematic diagram of the target sequence, PAM, and ssDNA at *Col4α5* gene locus. **(b)** Direct sequence of mutation on *Col4α5* mutant males. **(c)** PCR genotyping in *Col4α5* deficient rats. **(d)** Efficiencies of rat *Col4α5* gene editing with the rGONAD technology.

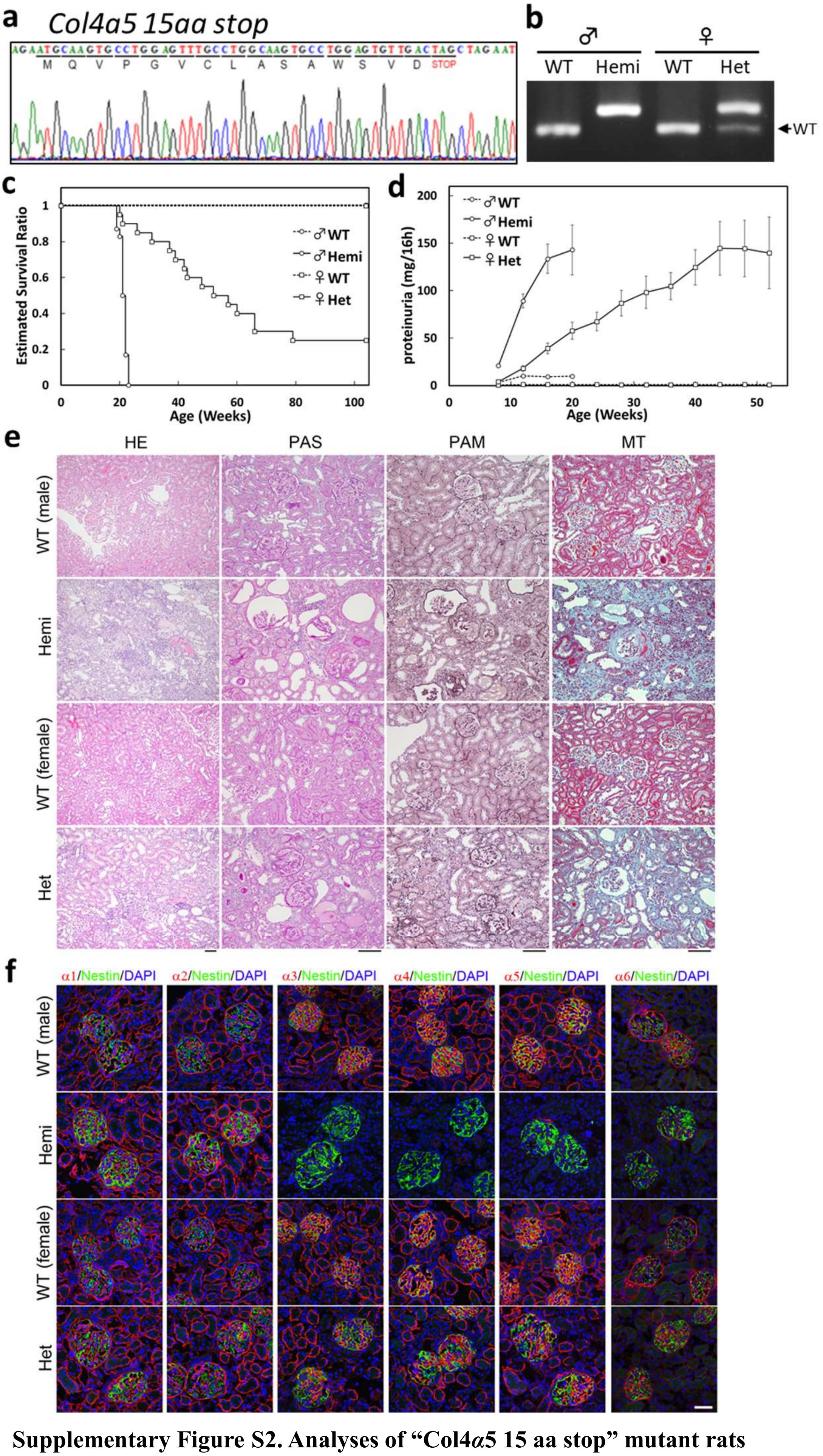

Supplementary Figure S2. Analyses of "Col4α5 15 aa stop" mutant rats

(a) Direct sequence of mutation. **(b)** PCR genotyping. **(c)** Estimated survival functions. **(d)** Proteinuria **(e)** Histological analyses at 20 weeks of age. **(f)** Type IV collagen distributions at 8 weeks of age. *Scale bars*, **(e)**: 100 μm, **(f)**: 50 μm.

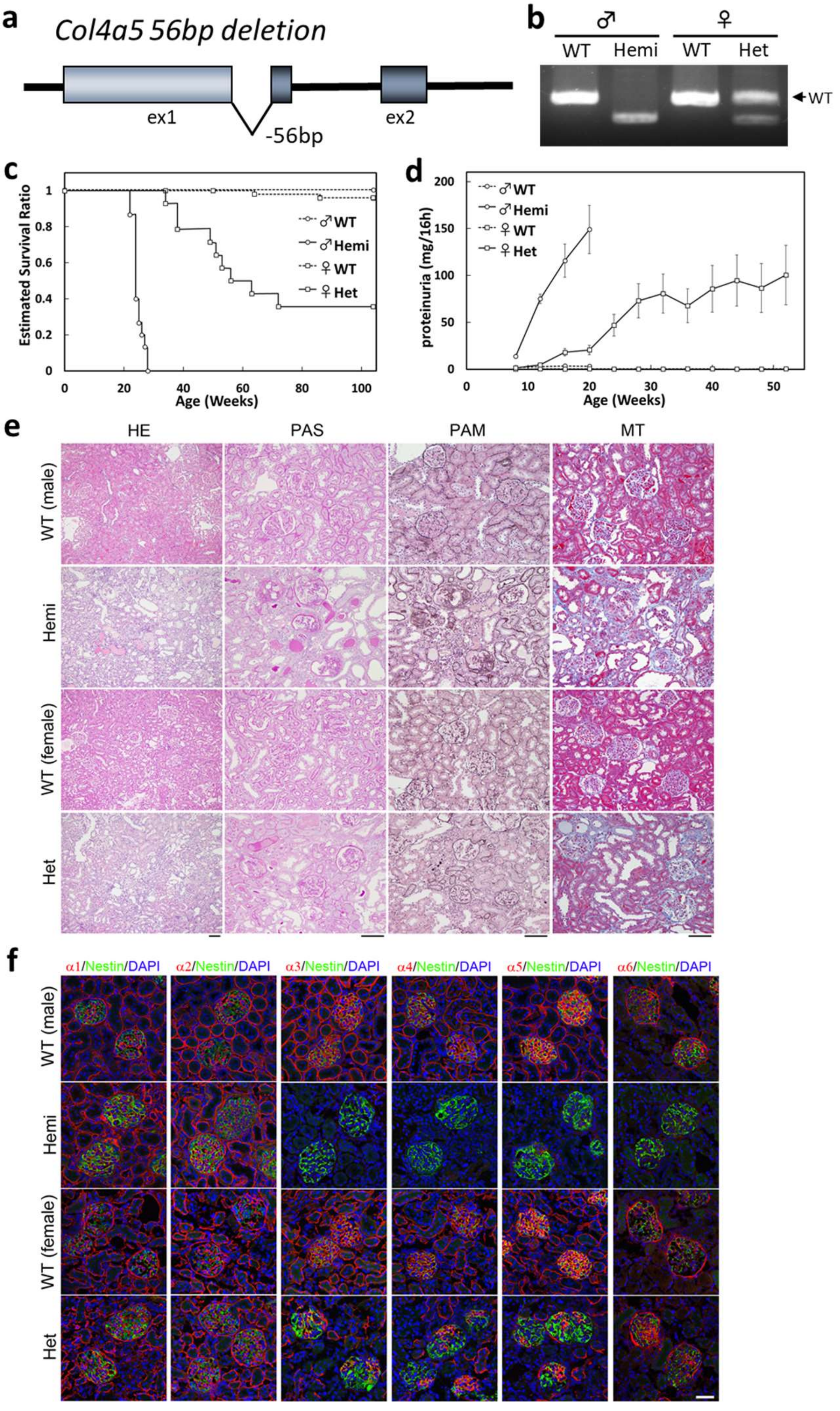

Supplementary Figure S3. Analyses of "*Col4α5* 56 bp deletion" mutant rats
(a) Schematic diagram of *Col4α5* 56 bp deletion. **(b)** PCR genotyping. **(c)** Estimated survival functions. **(d)** Proteinuria **(e)** Histological analyses at 20 weeks of age. **(f)** Type IV collagen distributions at 8 weeks of age. *Scale bars*, **(e)**: 100 µm, **(f)**: 50 µm.

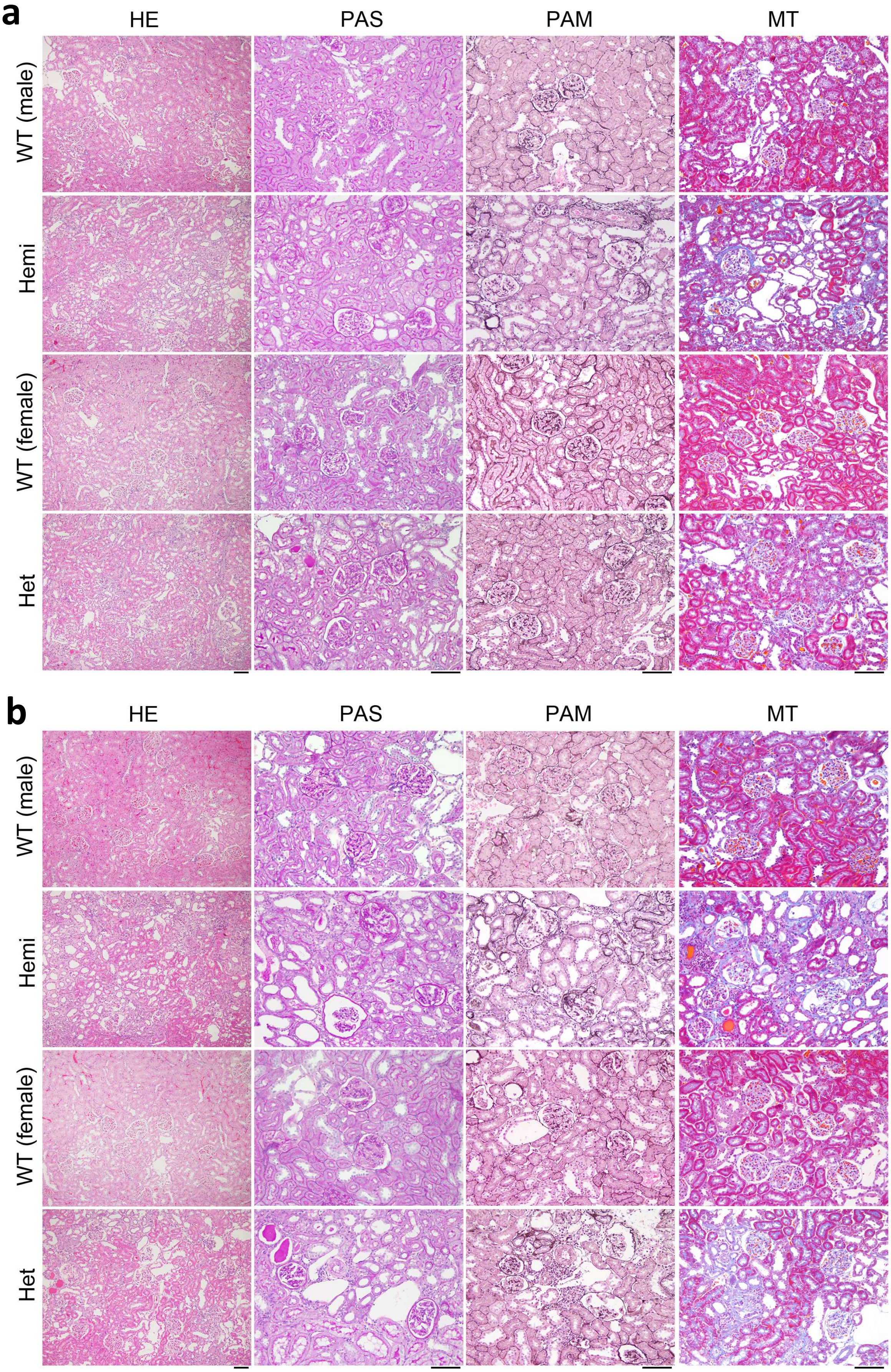

Supplementary Figure S4. Histological analyses of *Col4α5* deficient kidneys
Representative microscopic images in wildtype (WT) and *Col4α5* mutant (Hemi; hemizygous males, Het; heterozygous females) rats at 12 weeks **(a)** and 16 weeks **(b)** of age. These tissue sections were prepared and stained with hematoxylin and eosin (HE), periodic acid Schiff (PAS), periodic acid methenamine silver (PAM), and Masson trichrome (MT). *Scale bars*, 100 μm.

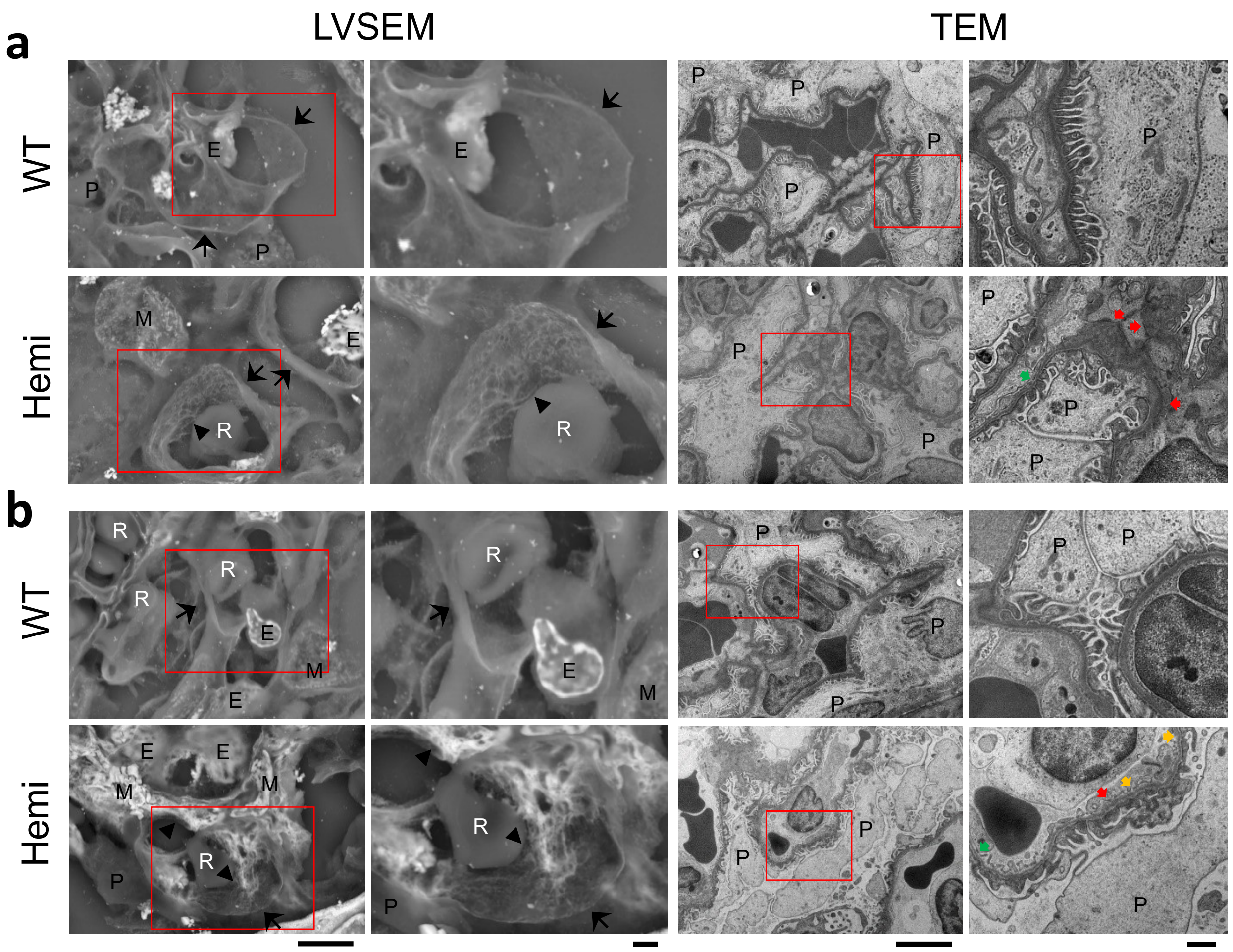

Supplementary Figure S5. Electron photomicrographs of glomerular basement membranes in *Col4α5* mutant rats

(a, b) Representative Low-vacuum scanning electron microscopy (LVSEM, left) and transmission electron microscopy (TEM; right) images in wildtype (WT) and *Col4α5* mutant (Hemi) males at 12 weeks **(a)** and 16 weeks **(b)** of age. Black arrowheads indicate the coarse meshwork structure of the GBM. Black arrows indicate cut side of the capillary walls. Red arrows indicate thickening of the GBM. Green arrows indicate thin patterns of the GBM. Yellow arrows indicate the splitting or fragmenting of the lamina densa. Red insets are revealed the higher magnification of left panels. E: Endothelial cells, M: Mesangial cells, P: Podocytes, R: Red blood cells. *Scale bars*, 5 µm (left), 1 µm (right).

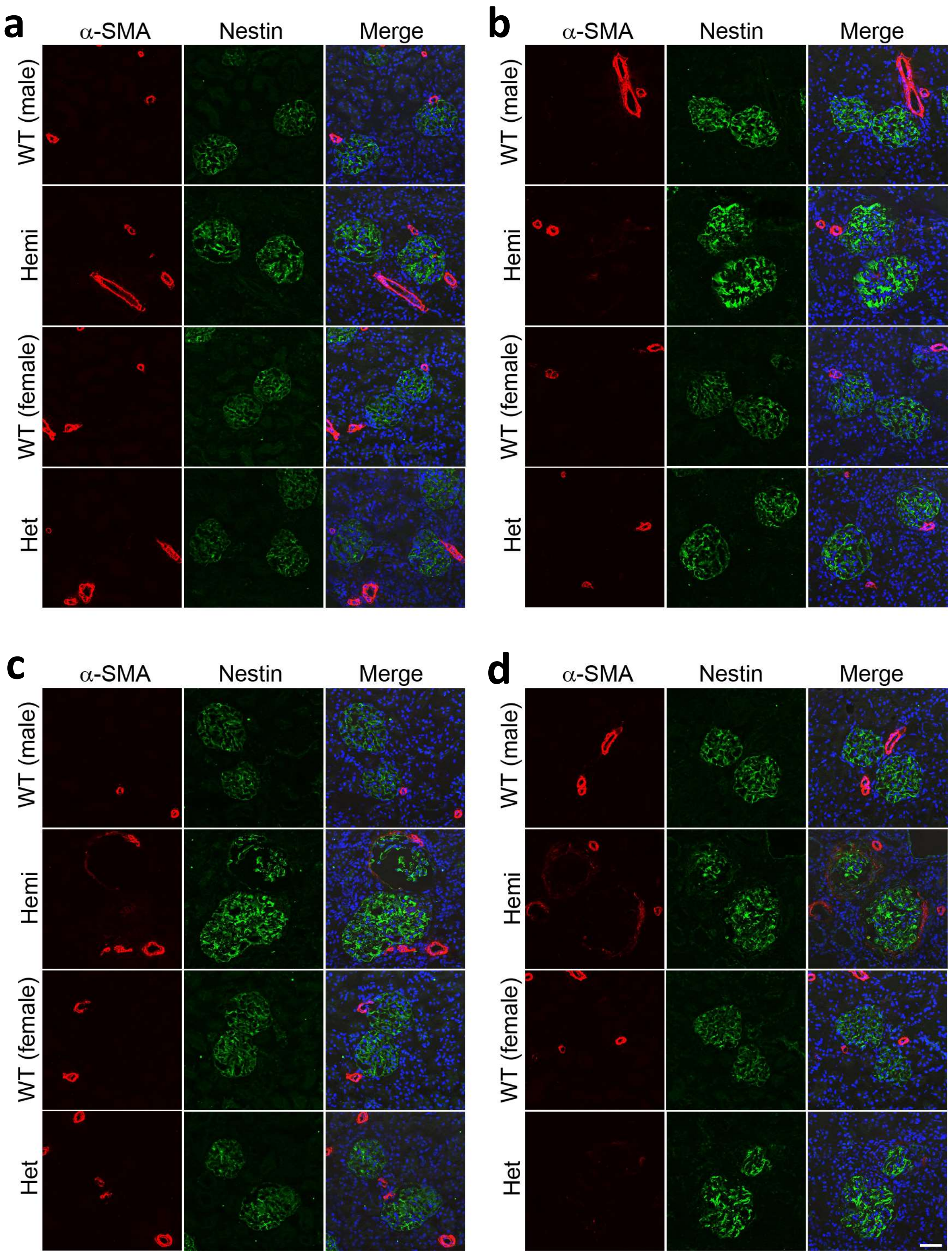

Supplementary Figure S6. Renal fibrosis in *Col4α5* deficient rats

(a-d) Immunostaining of kidney sections with α-SMA (red), Nestin (green; glomeruli), and DAPI (blue; nuclei) in wildtype (WT) and *Col4α5* mutant (Hemi; hemizygous males, Het; heterozygous females) rats from 8 **(a)**, 12 **(b)**, 16 **(c)**, to 20 **(d)** weeks of age. *Scale bars*, 50 μm.

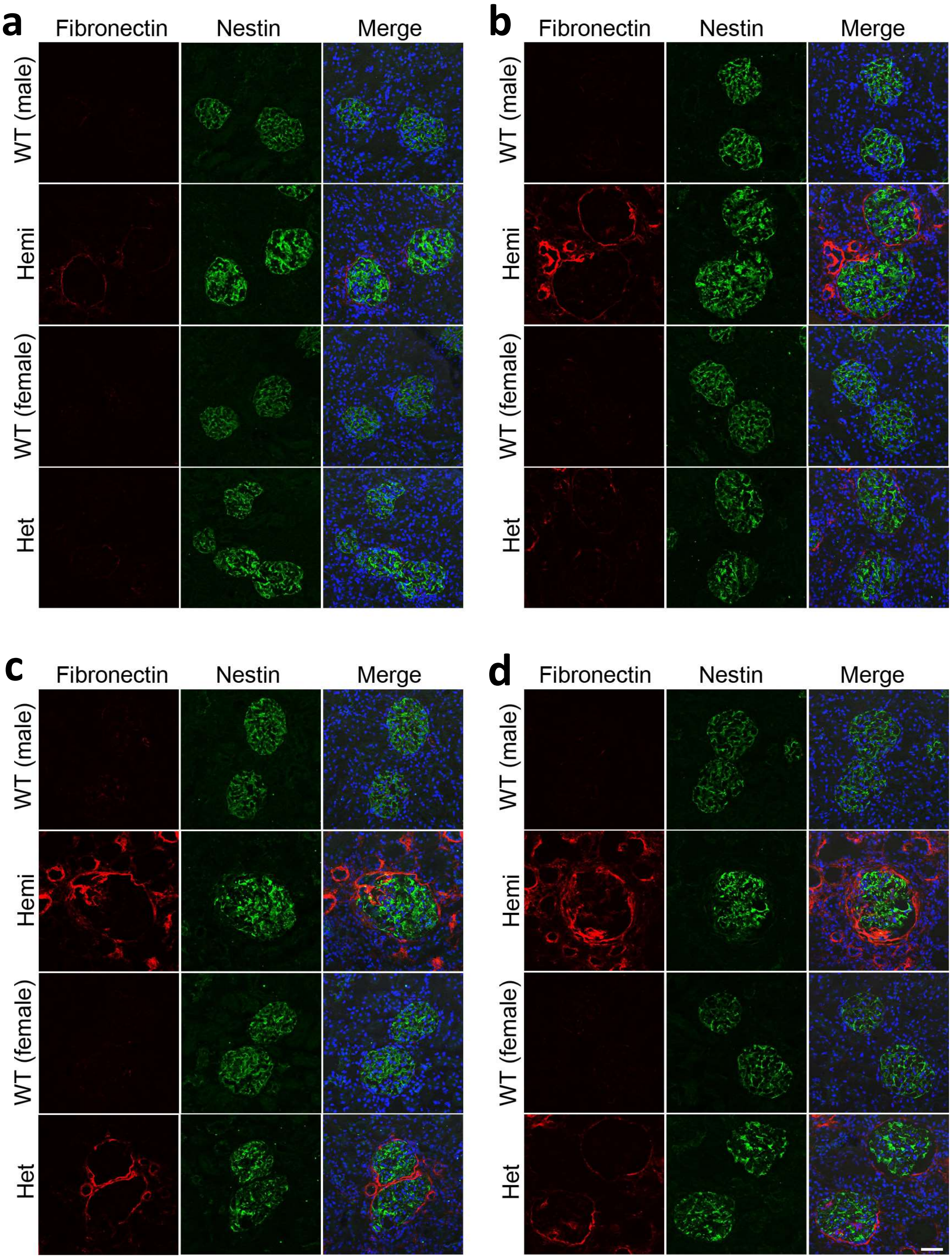

Supplementary Figure S7. Renal fibrosis in *Col4α5* deficient rats

(a-d) Immunostaining of kidney sections with fibronectin (red), Nestin (green; glomeruli), and DAPI (blue; nuclei) in wildtype (WT) and *Col4α5* mutant (Hemi; hemizygous males, Het; heterozygous females) rats from 8 **(a)**, 12 **(b)**, 16 **(c)**, to 20 **(d)** weeks of age. *Scale bars*, 50 μm.

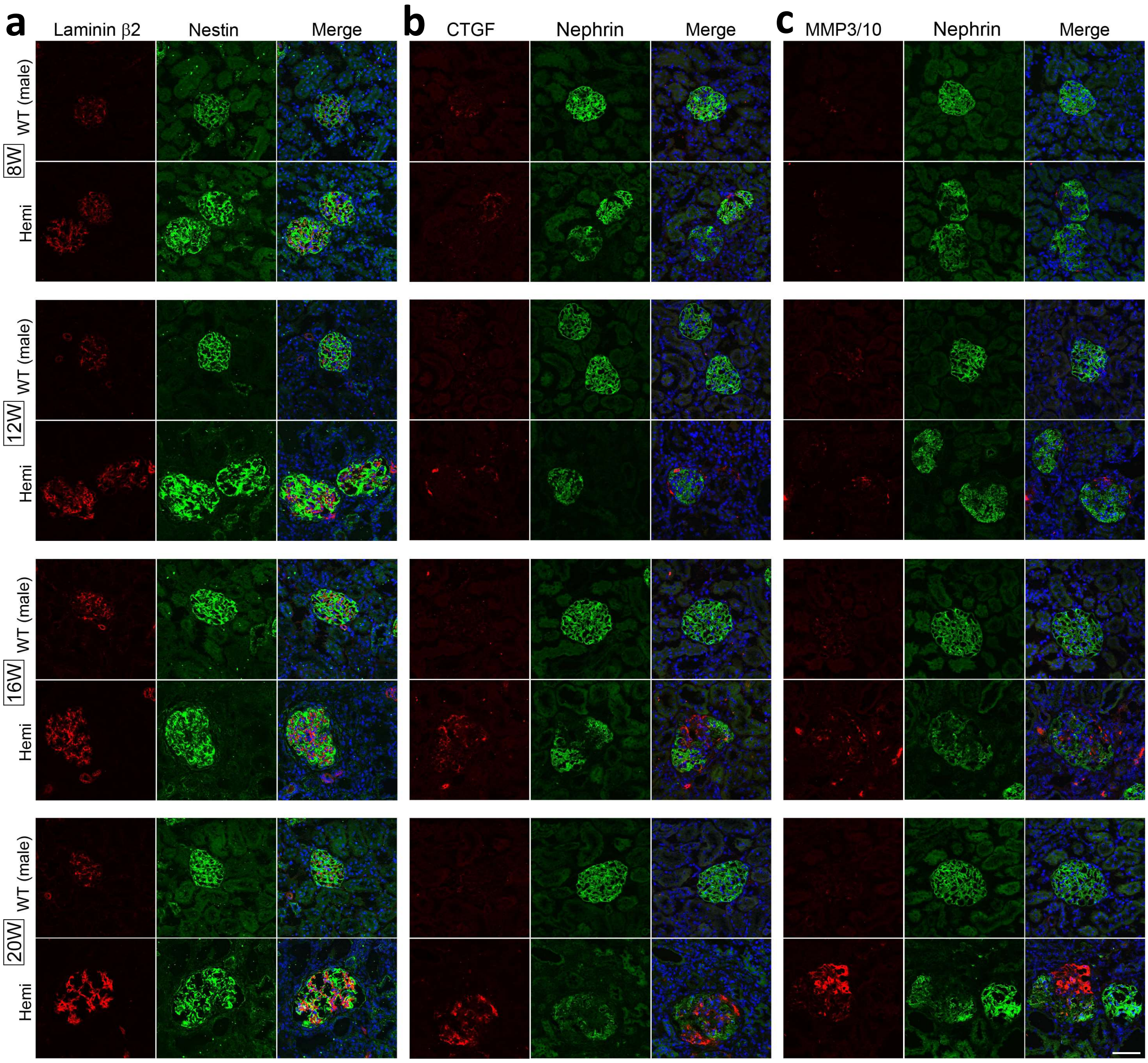

Supplementary Figure S8. Immunostaining of the GBM in *Col4α5* deficient kidneys
(a-c) Immunofluorescence analyses of the rat kidney sections with antibodies against **(a)**: Laminin β2 (red), Nestin (green; glomeruli); **(b)**: CTGF (red), Nephrin (green); **(c)**: MMP3/10 (red), Nephrin (green); and DAPI (blue; nuclei) in wildtype (WT) and *Col4α5* mutant (Hemi) male rats from 8, 12, 16, to 20 weeks of age. *Scale bars*, 50 µm.

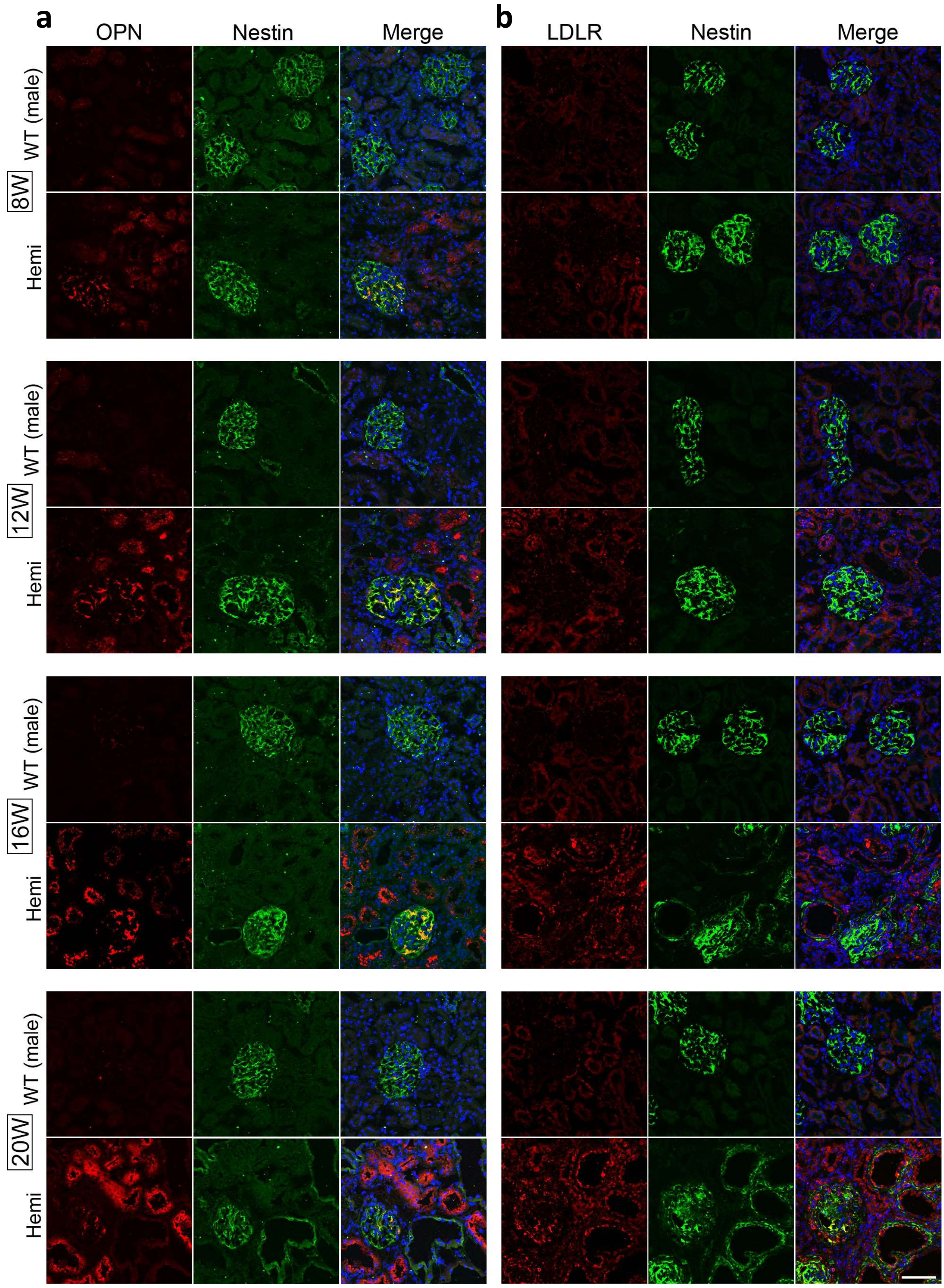

Supplementary Figure S9. OPN and LDLR expressions in *Col4α5* deficient rats
(a, b) Immunostaining of kidney sections with **(a)** OPN or **(b)** Low Density Lipoprotein Receptor (LDLR) (red), Nestin (green; glomeruli), and DAPI (blue; nuclei) in wildtype (WT) and *Col4α5* mutant (Hemi) male rats from 8, 12, 16, to 20 weeks of age. *Scale bars*, 50 μm.

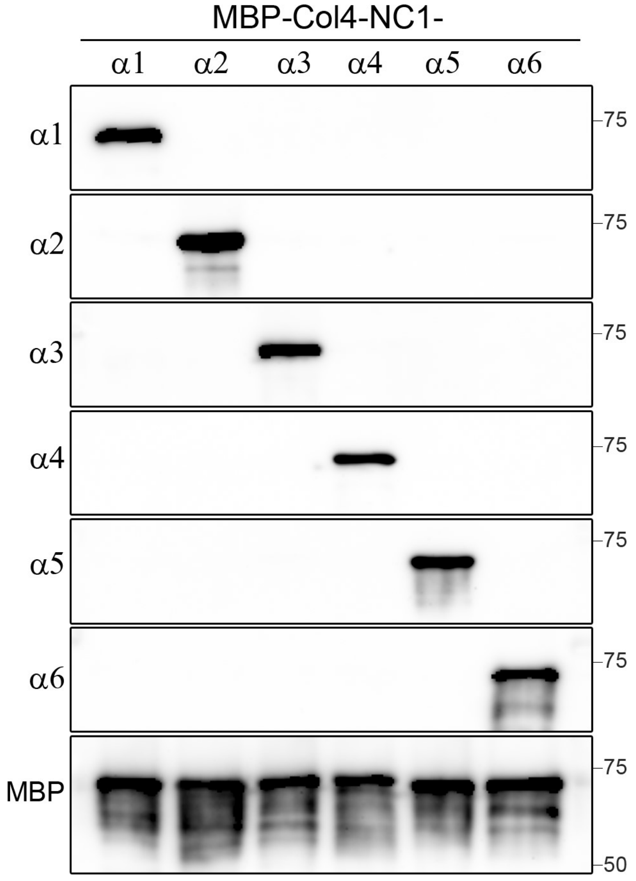

Supplementary Figure S10. Characterization of an antibody specifically recognized type IV collagen

Immunoreactivity was observed specifically with COL4A6 (α6), but not with other COL4 protein (α1-5). The type IV collagen α1-5 protein antibodies were also immunoreacted specifically with COL4 protein (α1-5) proteins, respectively.

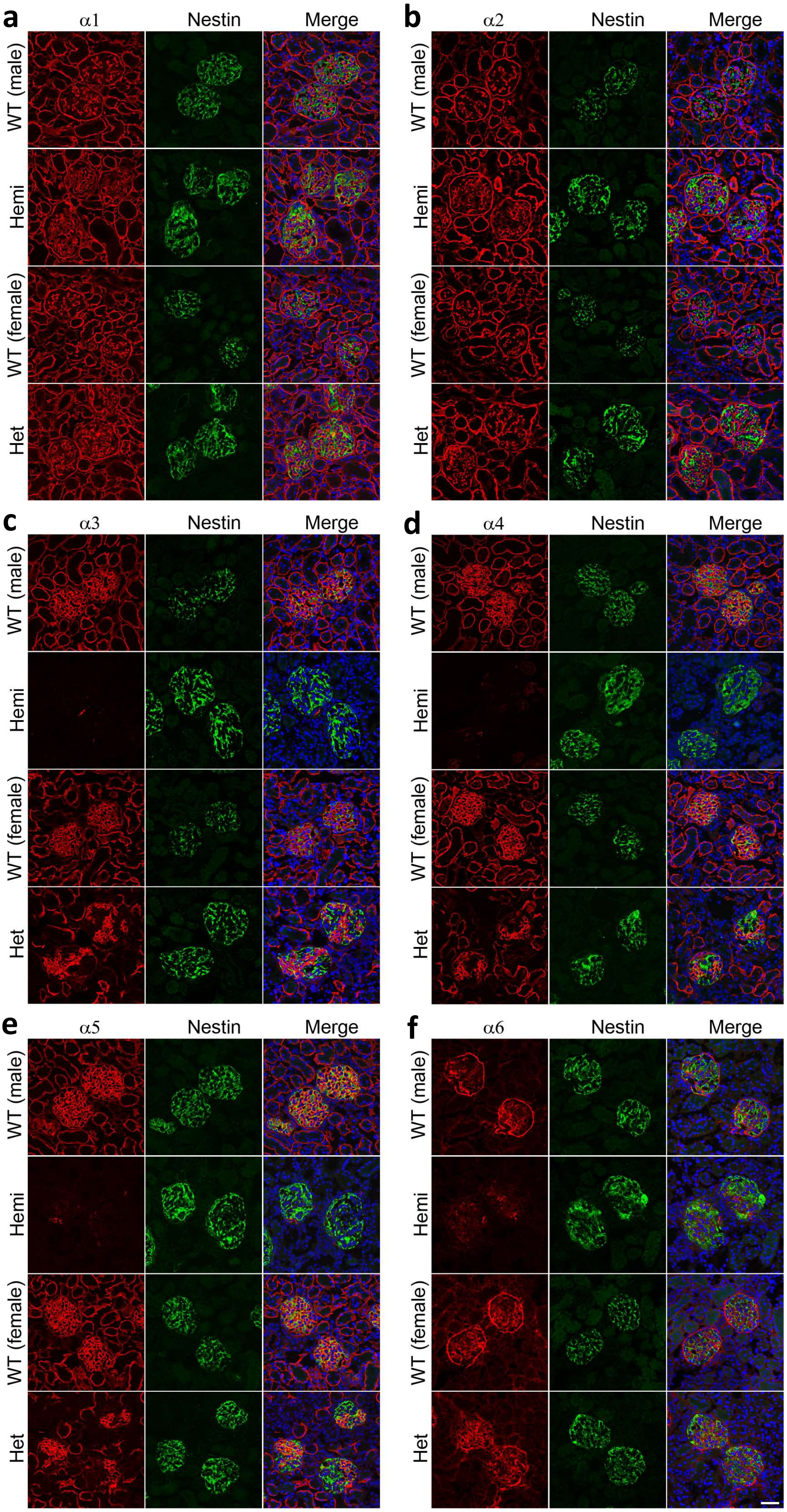

Supplementary Figure S11. Type IV collagen distributions in *Col4α5* deficient kidneys at 8 weeks of age

(a-f) Immunofluorescence analyses of kidney sections with antibodies against α1-6 (IV) (red), Nestin (green; glomeruli), and DAPI (blue; nuclei) in wildtype (WT) and *Col4α5* mutant (Hemi; hemizygous males, Het; heterozygous females) rats. *Scale bars*, 50 µm.

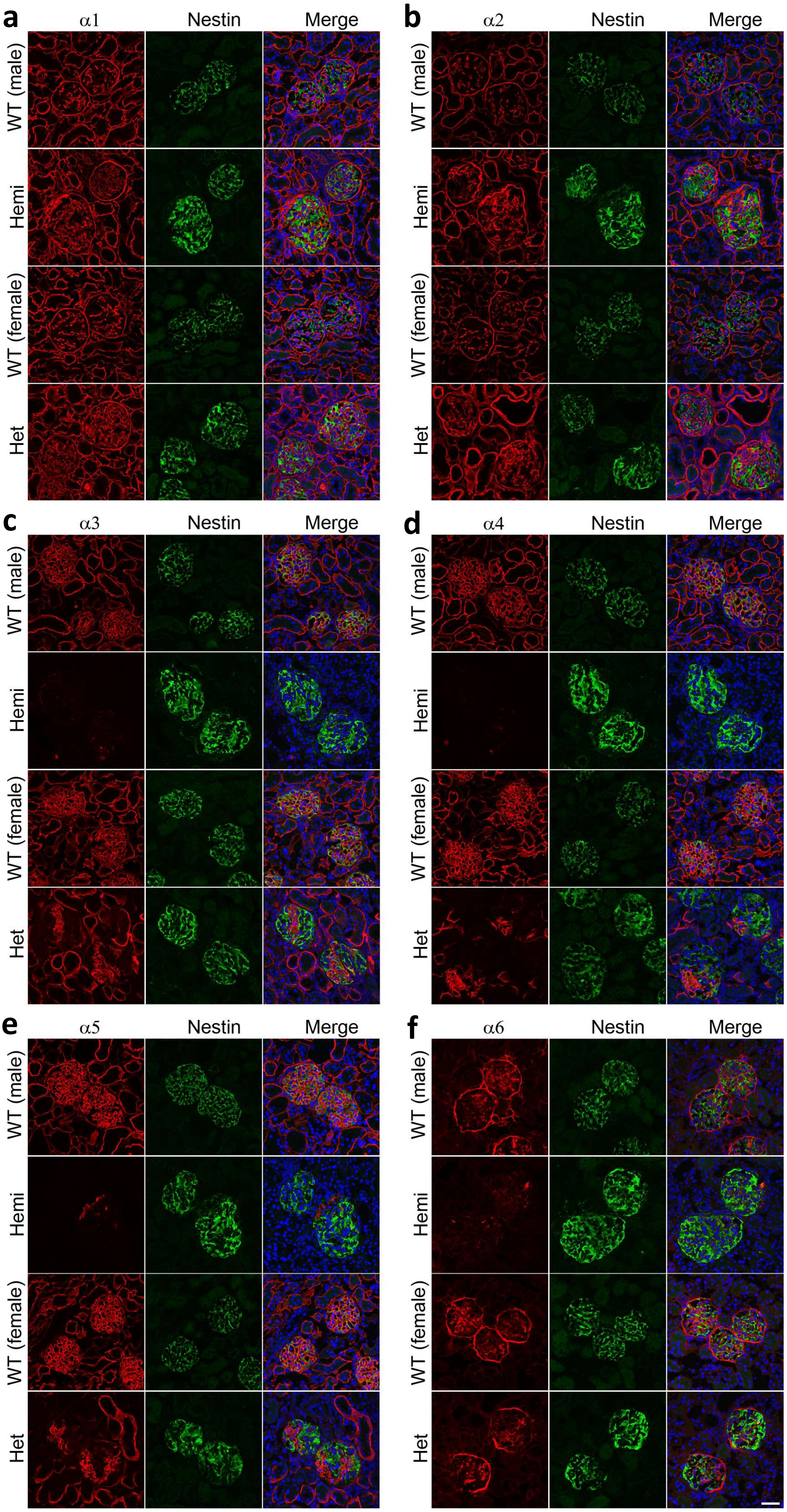

Supplementary Figure S12. Type IV collagen distributions in *Col4α5* deficient kidneys at 12 weeks of age

(a-f) Immunofluorescence analyses of kidney sections with antibodies against α1-6 (IV) (red), Nestin (green; glomeruli), and DAPI (blue; nuclei) in wildtype (WT) and *Col4α5* mutant (Hemi; hemizygous males, Het; heterozygous females) rats. *Scale bars*, 50 μm.

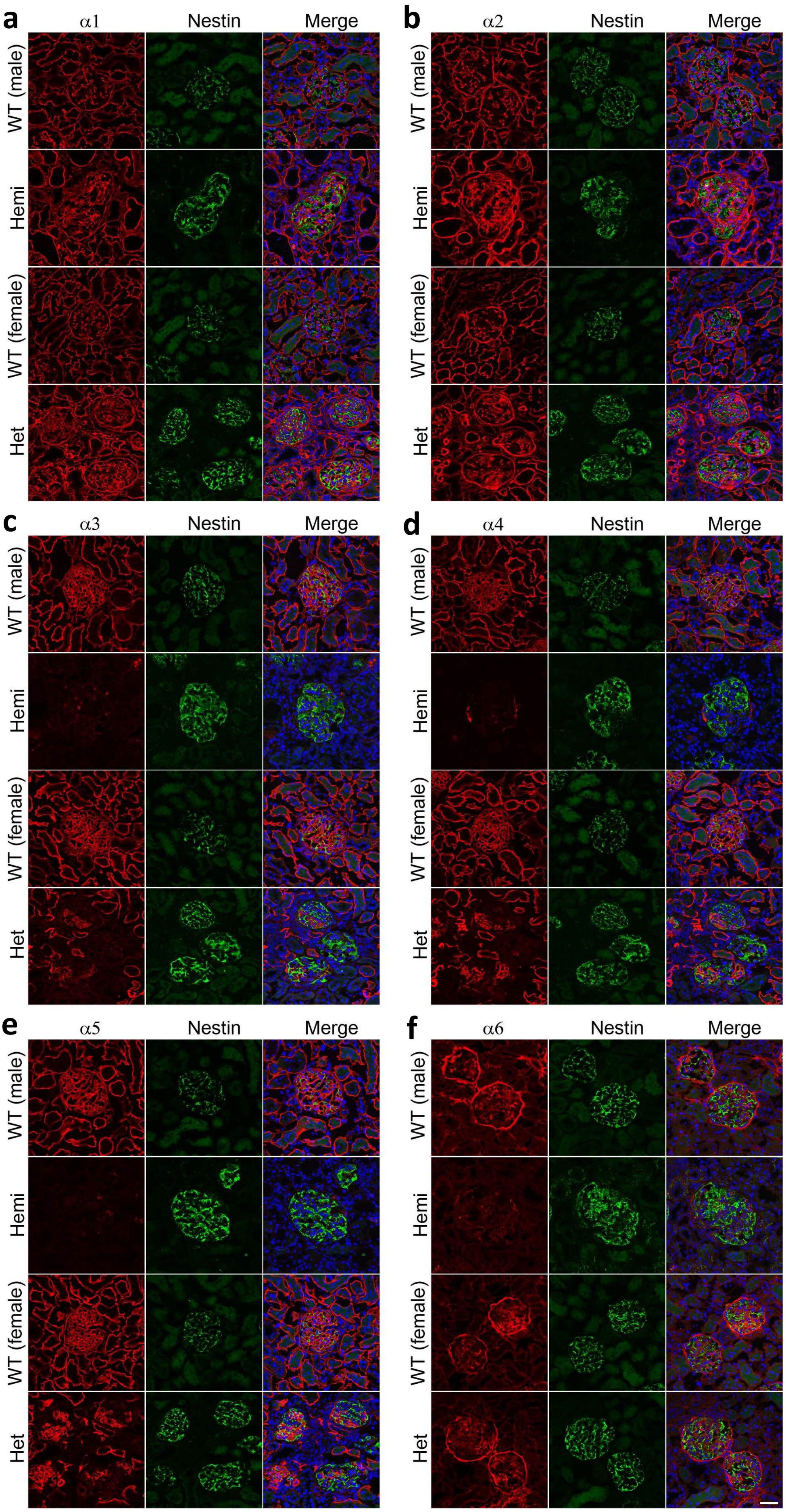

Supplementary Figure S13. Type IV collagen distributions in *Col4α5* deficient kidneys at 16 weeks of age

(a-f) Immunofluorescence analyses of kidney sections with antibodies against α1-6 (IV) (red), Nestin (green; glomeruli), and DAPI (blue; nuclei) in wildtype (WT) and *Col4α5* mutant (Hemi; hemizygous males, Het; heterozygous females) rats. *Scale bars*, 50 μm.

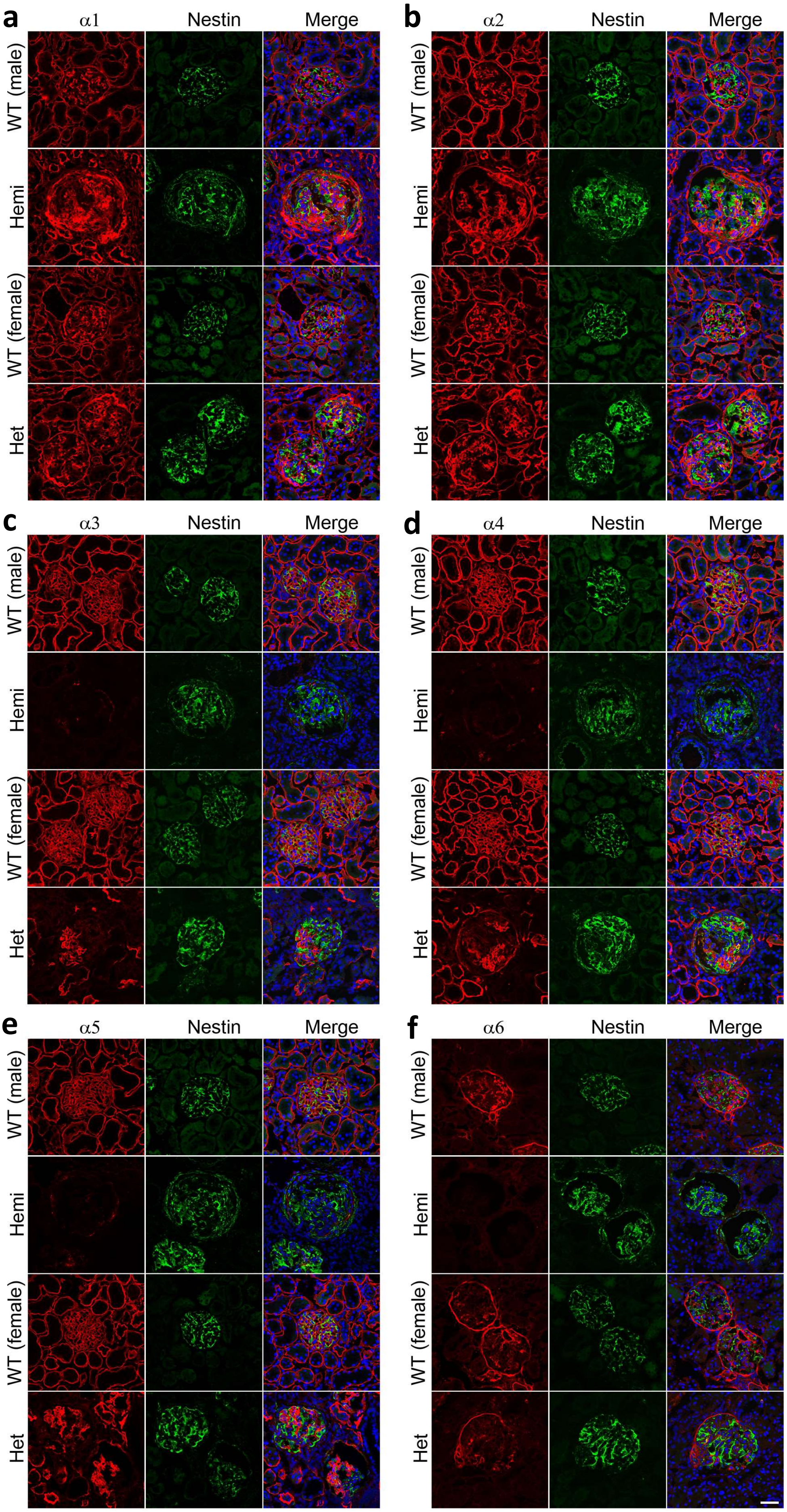

Supplementary Figure S14. Type IV collagen distributions in *Col4α5* deficient kidneys at 20 weeks of age

(a-f) Immunofluorescence analyses of kidney sections with antibodies against α1-6 (IV) (red), Nestin (green; glomeruli), and DAPI (blue; nuclei) in wildtype (WT) and *Col4α5* mutant (Hemi; hemizygous males, Het; heterozygous females) rats. *Scale bars*, 50 μm.

Figure 5 (d)

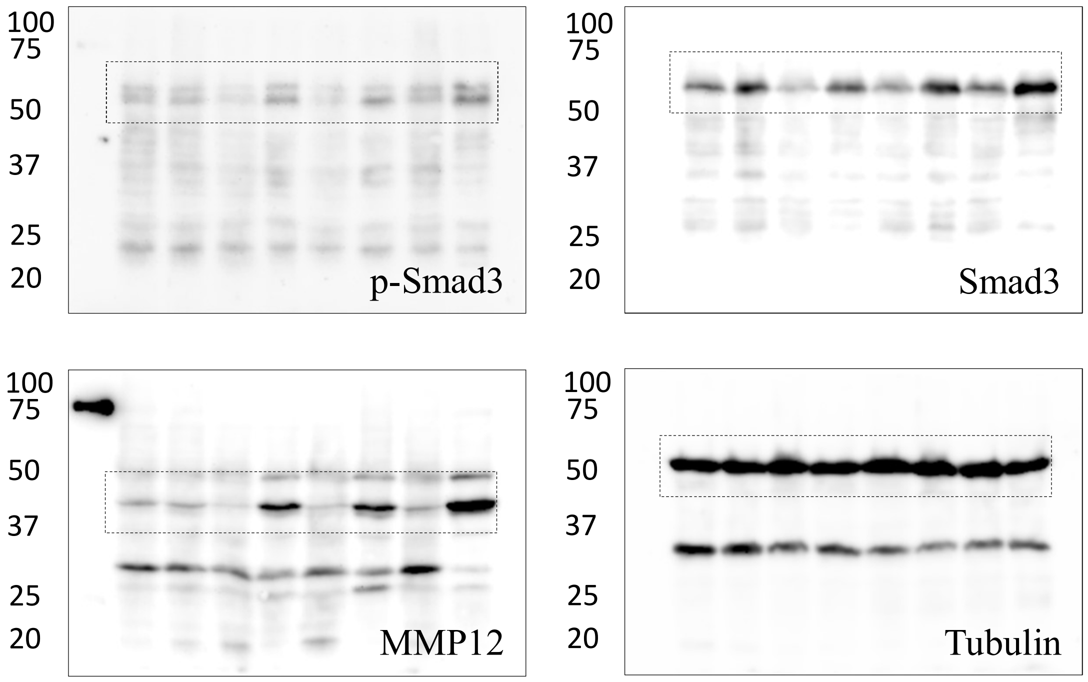

Figure 6 (c)

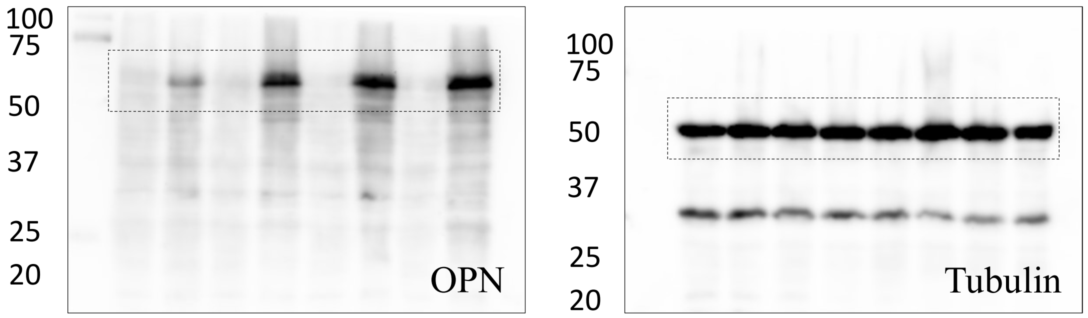

Figure 6 (d)

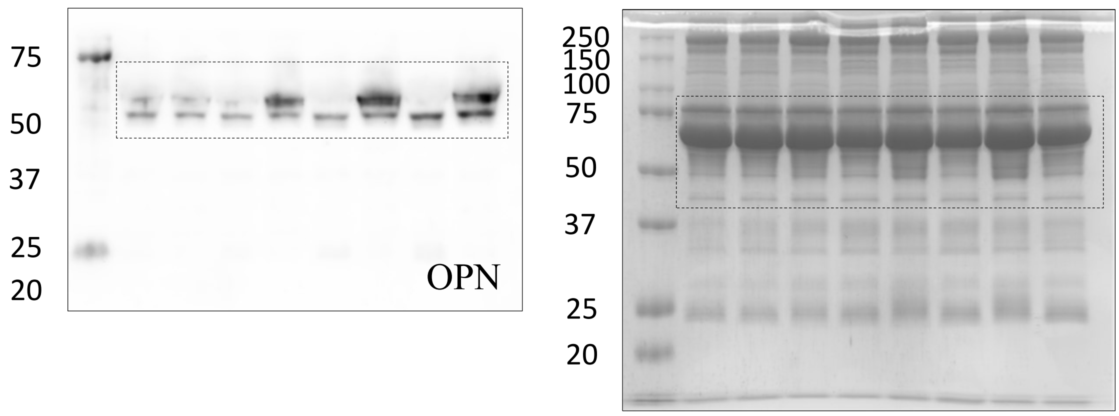

Supplemental Figure S15. Scan images for Western blotting

Figure 8

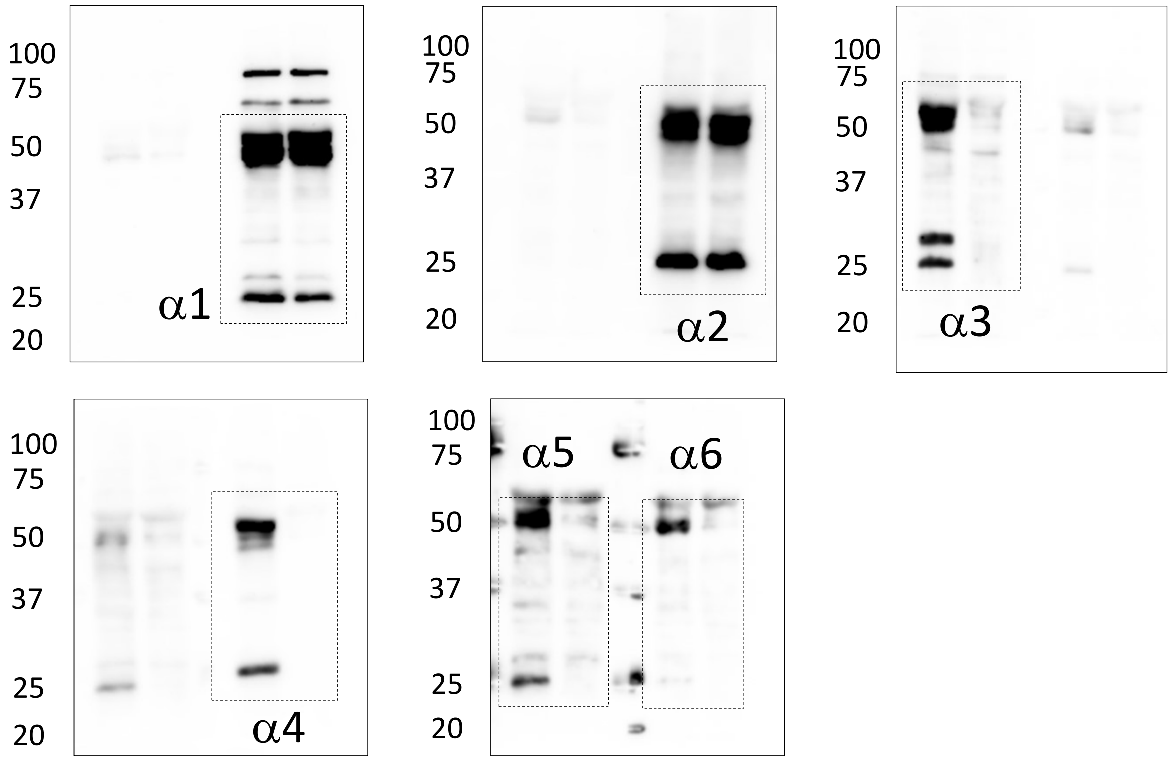

Figure S10

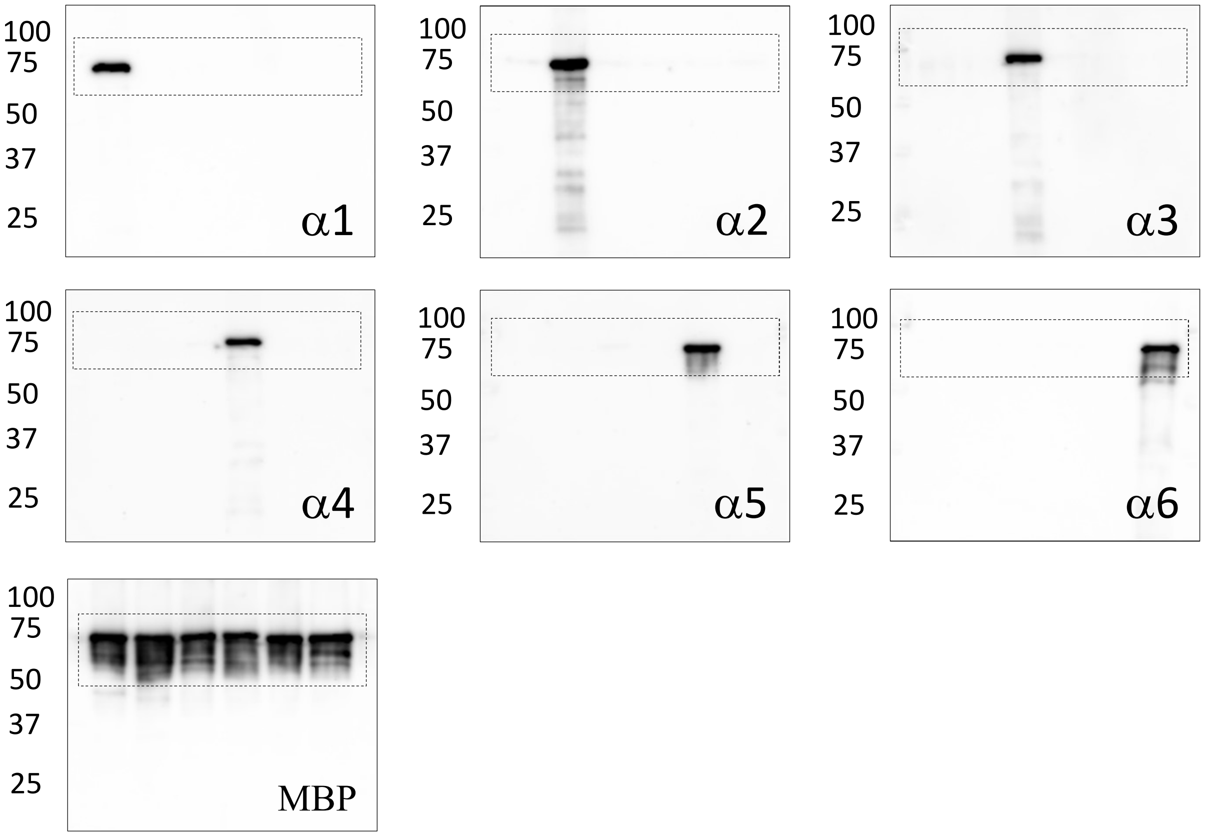

Supplemental Figure S15. Scan images for Western blotting

Figure S1

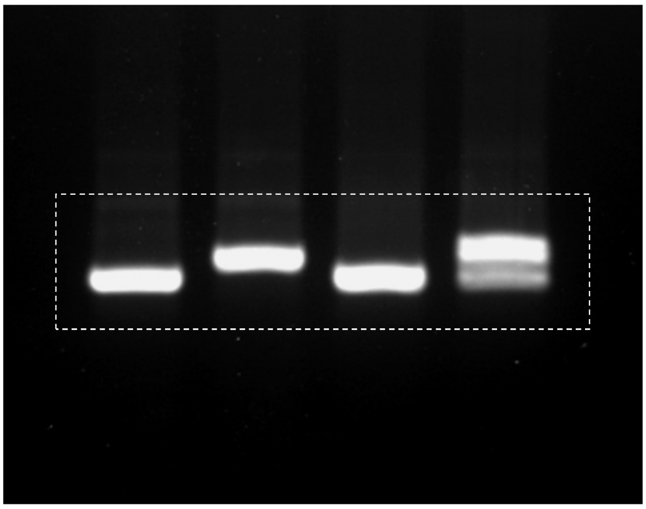

Figure S2

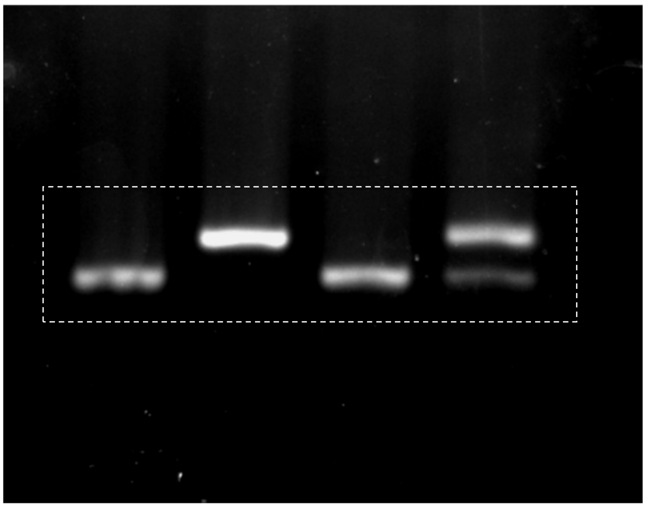

Figure S3

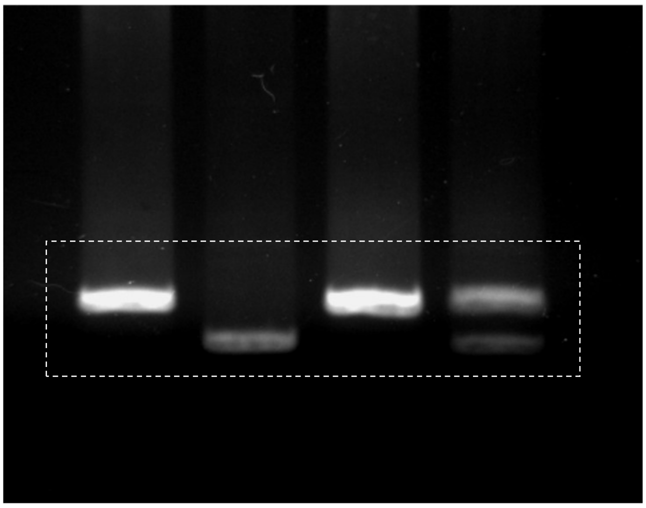

Supplemental Figure S16. Scan images of gel electrophoresis

Supplementary Table S1. Proteinuria in *Col4a5* deficient rats

	WT (male)		Hemi		WT (female)		Het	
	proteinuria (mg/16h)	n	proteinuria(mg/16h)	n	proteinuria (mg/16h)	n	proteinuria(mg/16h)	n
4W	0.1 ± 0.0	10	0.6 ± 0.1	16	0.1 ± 0.0	12	0.5 ± 0.1	11
6W	0.4 ± 0.1	10	6.2 ± 0.4	12	0.4 ± 0.1	8	2.0 ± 0.5	10
8W	2.7 ± 0.2	32	16.4 ± 0.9	57	0.7 ± 0.0	58	3.5 ± 0.3	42
12W	9.2 ± 0.7	35	77.0 ± 3.8	59	1.1 ± 0.0	59	14.2 ± 1.8	43
16W	8.9 ± 0.7	37	87.5 ± 6.8	59	1.2 ± 0.1	60	30.5 ± 3.8	45
20W	8.5 ± 0.5	35	100.1 ± 12.9	53	1.1 ± 0.0	58	42.9 ± 5.3	41
24W					1.1 ± 0.0	58	57.8 ± 7.8	40
28W					1.0 ± 0.0	57	85.3 ± 10.4	39
32W					1.0 ± 0.0	57	87.3 ± 10.9	39
36W					1.0 ± 0.0	51	124.1 ± 12.4	36
40W					1.0 ± 0.1	49	133.8 ± 15.9	34
44W					1.1 ± 0.1	47	157.2 ± 25.0	32
48W					1.2 ± 0.1	40	163.2 ± 30.5	30
52W					1.3 ± 0.1	40	175.7 ± 22.0	30

wildtype (WT), Hemizygous (Hemi), and Heterozygous (Het) mutant rats
